# Pregnancy and Assisted Reproductive Outcomes in Women with Systemic Lupus Erythematosus, Sjögren Syndrome and Antiphospholipid Syndrome: An Umbrella Review

**DOI:** 10.3390/jcm15072618

**Published:** 2026-03-30

**Authors:** Caixin Yue, Wanrong Huang, Jinbiao Han, Yuzhu Zhang, Xun Zeng, Rui Gao, Lang Qin

**Affiliations:** 1 Reproductive Medical Center, Department of Obstetrics and Gynecology, West China Second University Hospital, Sichuan University, Chengdu 610041, China; 2 Key Laboratory of Birth Defects and Related Diseases of Women and Children, Ministry of Education, West China Second University Hospital, Sichuan University, Chengdu 610041, China; 3 West China Lecheng Hospital of Sichuan University, Qionghai 571437, China; 4 Department of Laboratory Medicine, West China Second University Hospital, Sichuan University, Chengdu 610041, China; 5 Department of Obstetrics and Gynecology, West China Second University Hospital, Sichuan University, Chengdu 610041, China; 6 Meishan Women and Children’s Hospital, Meishan 620010, China; 7 Development and Related Diseases of Women and Children Key Laboratory of Sichuan Province, West China Second University Hospital, Sichuan University, Chengdu 610041, China

**Keywords:** systemic lupus erythematosus, Sjögren syndrome, antiphospholipid syndrome, pregnancy outcome, assisted reproductive outcome, umbrella review

## Abstract

**Objective:** Systemic lupus erythematosus (SLE), Sjögren syndrome (SS) and antiphospholipid syndrome (APS) are common autoimmune conditions in child-bearing aged women, but their influence on pregnancy and assisted reproductive outcomes remain controversial. We aimed to perform an umbrella review to summarize the current evidence to provide a reference for clinicians and future research. **Methods:** PubMed, Embase (Ovid) and Cochrane database were searched (inception to April 2025) for relevant publications. Study selection, data extraction, quality evaluation, evidence grading and data synthesis were completed independently by two authors. Odds ratio, relative risk or standardized mean difference with 95% confidence intervals were calculated. **Results:** Fourteen articles (51 meta-analyses) were included, to report the associations of SLE, primary SS (pSS), antiphospholipud antibodies (aPLs), primary APS (pAPS) and 6 maternal/8 fetal/5 assisted reproductive outcomes. SLE and pAPS significantly increased the risks of spontaneous abortion, total fetal loss, pregnancy-induced hypertension, premature delivery, small for gestational age, neonatal death and neonatal intensive care unit. SLE also decreased anti-Müllerian hormone level and significantly increased the risks of pre-eclampsia (PE), stillbirth, low birth weight (LBW) and neonatal one minute Apgar < 7. pSS significantly increased spontaneous abortion and LBW risks. Positive aPLs significantly increased the risk of miscarriage rate in assisted reproductive techenology (ART) and were also associated with total fetal loss, PE, intrauterine growth retardation and placental abruption. **Conclusions:** This review offers a thorough overview of the current evidence linking SLE, SS and APS to pregnancy and assisted reproductive outcomes. It identifies existing gaps and proposes future research directions.

## 1. Introduction

Autoimmune diseases are conditions in which the immune system erroneously targets and attacks the body’s antigens, resulting in organ damage and dysfunction [1]. As reported, autoimmune diseases affect 10% of the global population, with the percentage steadily rising, resulting in considerable economic and health challenges [2]. Notably, many autoimmune diseases present significant differences in incidence and prevalence between males and females. The most obvious among them is that the male-to-female ratios of systemic lupus erythematosus (SLE), Sjögren syndrome (SS) and antiphospholipid syndrome (APS) have reached approximately 1:9, 1:14 and 1:3.5 respectively [3,4,5]. The pronounced gender differences in the incidence of SLE, SS and APS suggest that these conditions may have specific influences on certain physiological processes unique to women, such as pregnancy.

In the last two decades, although maternal and neonatal mortality have effectively decreased around the world, the incidence of many other fertility and pregnancy disorders, such as infertility, spontaneous abortion, pre-eclampsia, stillbirth and premature delivery, still heavily affects female reproductive health [6,7,8]. There were also more and more infertility patients got pregnany by assisted reproductive technology (ART) around the world [9,10], but the failure assisted reproductive outcomes bring significant adverse effects on females who expected to get pregnant. Women of child-bearing age are a susceptible population for various autoimmune diseases, especially for SLE, SS and APS [11,12]. SLE is a potentially fatal, chronic, multisystem autoimmune disorder that typically affects women between puberty and menopause [13]. It is acknowledged that SLE increased the risk of miscarriage, pre-eclampsia and fetal congenital heart block (CHB) [14], but the effects of SLE on assisted reproductive outcomes and other pregnancy outcomes have not yet reached a consensus. SS is a systemic autoimmune disease that mainly affects the exocrine glands [15]. Anti-SSA/SSB antibodies the most specific autoantibodies of SS [16]. SS was previously regarded as a increased risk of fetal CHB because of the specific pathogenic role of anti-SSA/SSB antibodies [17,18], but recently, its influence on various pregnancy outcomes and assisted reproductive outcomes has been focused on [19,20]. APS is an autoimmune disease characterized by the presence of circulating antiphospholipid antibodies (aPLs) [21]. Stillbirth, severe pre-eclampsia and placental insufficiency are acknowledged manifestations of APS [22]. However, the association between recurrent early miscarriage and APS is often considered but least defined. Some studies have indicated that patients with positive aPLs have a higher miscarriage rate [23], but other studies have shown similar aPLs positive rates in recurrent miscarriage patients and controls [24]. Moreover, the influence of APS/aPLs on assisted reproductive outcomes has not yet been established consistently. The uncertainties in the above-mentioned clinical associations hamper the establishment of individual management strategies for women with these conditions.

In recent years, some systematic review and meta-analyses based on the observational studies provided more evidence on the associations between SLE, SS, APS and various pregnancy and assisted reproductive outcomes [20,23,25,26,27], but these studies always focused on limited exposure or limited outcome and did not bring the systematic understanding on this problem. Herein, we performed an umbrella review to summarize the current evidence from systematic review and meta-analyses regarding the association between SLE, SS, APS and pregnancy and assisted reproductive outcomes, to provide a reference for clinicians and future research.

## 2. Methods

This umbrella review was conducted in accordance with the Joanna Briggs Institute (JBI) umbrella review methodology and the PRIOR (Preferred Reporting Items for Overviews of Reviews) checklist [28,29]. The protocol was registed in PROSPERO (number: CRD420251104552).

### 2.1. Search Strategy and Study Selection Criteria

We searched PubMed, Embase (Ovid) and the Cochrane database of systematic reviews for all relevant systematic review and meta-analyses of observational studies from database inception to April 2025. All authors collaboratively formulated the search strategy. The search terms of interested autoimmune conditions were set as “systemic lupus erythematosus”, “Sjögren syndrome”, “anti-SSA antibody”, “anti-SSB antibody”, “antiphospholipid antibodies” and “antiphospholipid syndrome”, as well as related words in databases. The search terms of pregnancy and assisted reproductive outcomes were complicated and confirmed by all authors, and we also asked other experts in this field to provide more references. In short, the detailed pregnancy outcomes include various maternal outcomes such as spontaneous abortion, fetal loss, pregnancy induced hypertension (PIH), pre-eclampsia (PE), gestational diabetes mellitus (GDM) and placental abruption (PA), as well as various fetal outcomes such as stillbirth, intrauterine growth retardation (IUGR), preterm delivery, small for gestational age (SGA), low birth weight (LBW), stillborn/neonatal death, neonatal intensive care unit (NICU) rate and Apgar score; the objective indicators of fertility include anti-Müllerian hormone (AMH) levels, ovarian reserve and infertility; the detailed indicators of assisted reproductive outcomes include clinical pregnancy rate (CPR), biochemical pregnancy rate, miscarriage rate and live birth rate (LBR). The detailed search strategy in PubMed, Embase (Ovid) and the Cochrane database of systematic reviews are shown in Tables S1–S3, respectively. In addition, we hand-searched and reviewed the references of the identified papers to avoid the loss of important literature.

The retrieved literature was then evaluated based on the inclusion and exclusion criteria to select eligible studies. The inclusion and exclusion criteria were confirmed by all authors. Titles and abstracts were first assessed by authors (C.Y. and W.H.) independently to screen the eligible studies. Then, a thorough examination of the full texts was conducted independently by four authors (C.Y. and W.H and R.G. and J.H.) to exclude non-qualifying works. In cases of differing opinions, discussions were held with the third author (X.Z.) until a consensus was reached.

### 2.2. The Inclusion and Exclusion Criteria

Systematic review and meta-analyses of observational studies that assessed the associations between SLE, SS, APS, anti-SSA/SSB antibodies, aPLs and pregnancy outcomes, fertility and assisted reproductive outcomes were included. Only studies published in English were included. The participants considered were women of child-bearing age, both pregnant or pnon-pregnant. If a meta-analysis is based on cohort studies, the exposure group must be women of reproductive age who possess SLE, SS, positive anti-SSA/SSB antibodies, positive aPLs or diagnosed APS, the outcomes must contain at least one indicator of pregnancy outcome, fertility or assisted reproductive outcome. If a meta-analysis is based on case-control or cross-sectional studies, the case group should be women with infertility, ovarian reserve dimmish, ART failure or adverse maternal or fetal outcomes, and the control group should be women without these conditions; the exposure should include at least one of SLE, SS, positive anti-SSA/SSB antibodies, positive aPLs and diagnosed APS. Systematic review and meta-analyses containing interventional studies, studies lacking sufficient data for re-analysis, studies without control group and Mendelian randomization studies were excluded. We also exclude studies involving women with other reproductive diseases, such as polycystic ovary syndrome and thyroid diseases.

### 2.3. Data Extraction

Data extraction was completed independently by two authors (C.Y. and R.G.) using previously established data extraction table. The extracted information includes the first author, publication year, title, review objectives, database searched, additional information source (e.g., grey literature), included meta-analyses, number of studies included, included study types, definition of exposures, definition of case (for meta-analyses based on case-control or cross-sectional studies), definition of outcomes (for meta-analyses based on cohort studies), data synthesis method, quality assessment tool, quality of the primary studies, methods for sensitivity analysis, methods of assessing publication bias. In addition, we collected the heterogeneity of the included meta-analysis by *I*^2^ statistics, which greater than 50% was regarded as considerable heterogeneity. We also identified outlier studies for included meta-analyses, when the confidential interval (CI) of one primary study does not overlap with the CI of the pooled effect, this outlier study should be removed to re-calculate the pooled estimate.

### 2.4. Quality Evaluation

The methodological quality of the included articles were evaluated according to AMSTAR2 by two authors (C.Y. and W.H.) independently. In case with differing opinions, we discussed and resolved them with the third author (L.Q.). The evaluation system consists of 16 items, of which 7 are cricial for the outcomes: items 2, 4, 7, 9, 11, 13, and 15 [30]. The assessment results of every item are categorized into four levels: high, medium, low, and very low. During assessments, if more than three crucial items fail to match the standards, the article is deemed of exceedingly low quality and will be excluded from the final analysis.

### 2.5. Evidence Grade

We classified the included meta-analyses into four categories according to the strength and robustness of the evidence: convincing (class I), highly suggestive (class II), suggestive (class III), weak (class IV), and not significant (NS). The grading process was jointly completed by the two authors (W.H. and J.H.). In summary, the criteria of convincing include *p* < 10^−6^, >1000 cases, *p* of the largest study < 0.05, and *I*^2^ < 50%, the criteria of highly suggestive include *p* < 10^−6^ and >1000 cases, the criteria of suggestive include *p* < 10^−3^ and >1000 cases, and the weak evidence refer to only *p* < 0.05.

### 2.6. Overlapping Calculation

When multiple articles examine the same autoimmune condition and the same outcome, it is essential to create an overlap matrix and to calculate the corrected covered area (CCA) to evaluate the degree of duplication of the original studies [31]. The formula is CCA = (*n* − r)/(rc − r), where *n* represents the total original studies included in the systematic review (including duplicates), r signifies the total number of original studies included after deduplication, and c represents the number of studies incorporated in this research for the systematic review. A CCA of 0–5% represents slight overlap, 6–10% indicates moderate overlap, 11–15% means signifies high overlap, and over 15% denotes extremely high overlap. If CCA = 0%, all the original studies data will be extracted for summary analysis. If CCA less than 10% but more than 0%, the original studies data in the included meta-analysis needs to be re-extracted for analysis but exclude the overlapping original articles. If CCA equal or more than 10%, we select the one with a higher quality according to AMSTAR2, or the most recently published, or the one encompassing a greater number of original studies, or with a larger sample size [32].

### 2.7. Statistical Analysis

If there was only one meta-analysis included for the association between one autoimmune condition and one outcome, odds ratio (OR), relative risk (RR) or standardizedmean difference (SMD) were selected according to the original meta-analysis and we did not re-calculated it. If we need to reanalyzing the included studies, we selected OR or RR according to the included study types. If the re-analyzed meta-analysis only included cohort studies, RR was selected, on the contrary, OR was selected. SMD was used to compare the quantitative index between two groups. The heterogeneity assessment in this study was conducted using *I*^2^ statistics. An *I*^2^ of < 25% was considered as low-level heterogeneity, 25%~50% was considered as moderate level and >50% was considered as high level. Random-effects models were employed when *I*^2^ ≥ 50% was observed; otherwise, fixed-effects models were applied. Funnel plots were used to assess publication bias. A symmetrical distribution of points on the funnel plot indicated the absence of significant publication bias. Conversely, an asymmetrical distribution suggested the presence of publication bias. Statistical analyses were performed using R software (version 4.3.1; R Foundation for Statistical Computing, Vienna, Austria).

## 3. Results

### 3.1. Basic Information of Included Articles

We initially retrieved 280 articles according to the search strategy mentioned previously. After removing 39 duplicates, we screened the titles and abstracts of the remaining 241 studies and excluded 212 based on initial criteria. Subsequently, the full texts of the remaining 29 articles were assessed against the inclusion and exclusion criteria, resulting in the exclusion of 13 articles that did not meet these criteria. Based on the AMSTAR2 score, we excluded 2 articles due to their extremely low quality, the results of quality assessment in these articles are shown in Table S4. Finally, fourteen articles were included [26,27,33,34,35,36,37,38,39,40,41,42,43,44].

**Figure 1:**
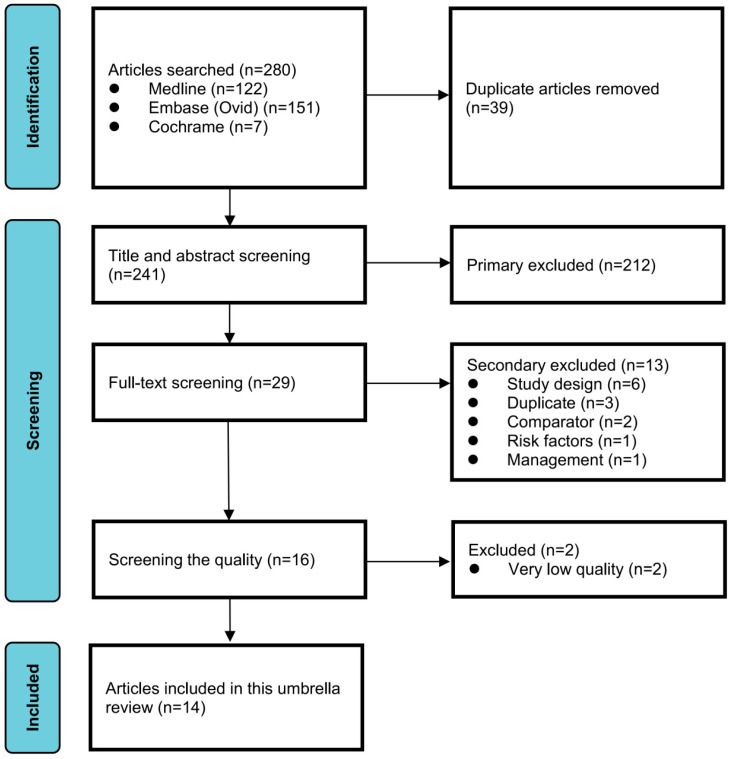
Flow chart of studies selection and inclusion.

Six articles explored the associations between SLE and pregnancy and assited reproductive outcomes. Two articles investigated the association between primary SS (pSS) and pregnancy and assited reproductive outcomes. Five articles focused on the association between positive aPLs and pregnancy and assited reproductive outcomes. Only one article explored the association between primary APS (pAPS) and maternal and fetal outcomes. There was no studies investigating the positive anti-SSA/SSB antibodies and pregnancy and assisted reproductive outcomes. Ten of 14 articles performed quality assessment for original studies by NOS scale. The detailed information of included articles are shown in Table 1. The flowchart of article selection and inclusion are presented in Figure 1.

### 3.2. Characteristics of Included Meta-Analyses

There were 51 meta-analyses in the included articles. Fourty-three of included meta-analyses performed sensitivity analyses, mainly by changing the analysis models or exclude each study. Eight of included meta-analyses did not perform publication bias analysis because the number of included original studies was small. For included meta-analyses with publication bias analysis, only one showed significant publication bias. All the 51 included meta-analyses were evaluated for heterogeneity by *I*^2^ statistics. Among them, 15 meta-analyses had *I*^2^ more than 50%, indicating high heterogenicity. Two included meta-analysis had outlier primary studies.

The grade of evidence of included meta-analysis ranged from suggestive (class III) to weak (class IV), and some showed NS. We found that there was no I and II, mainly because the *p*-value of the included meta-analyses did not reach *p* < 10^−6^. The characteritics of included meta-analyses are shown in Table 2.

### 3.3. Overlapping Analyses

The overlap range between the autoimmune conditions and the outcomes varies between 0 and 50% in this umbrella review. For SLE, there is an overlap with spontaneous abortion, PE, GDM, premature delivery, SGA, LBW, NICU and One minute Apgar < 7, with CCA of 0%, 30.76%, 50%, 6.25%, 0%, 0%, 0% and 0%. According to the previously established standards, data from the original studies concerning spontaneous abortion, SGA, LBW, NICU and One minute Apgar < 7 were all extracted for the final analysis (Figures S1, S2 and S5–S12), while for PE and GDM, one article was selected for the final analysis. Concerning premature delivery, all original articles from overlapping literature were re-extracted; however, data from duplicate publications were excluded from the integrated analysis (Figures S3 and S4). For pSS, two meta-analyses showed overlap concerning premature delivery, with a CCA of 16.67%. Based on the standards, one of these articles was selected for final analysis. For positive aPLs, there is an overlap with PE, IUGR, CPR in ART, LBR in ART and miscarriage in ART, with CCA of 26.92%, 20.83%, 35.71%, 40% and 50%. Only one article was selected for the final analyses. For pAPS, there was no overlap found. The detailed information are shown in Table 3 and Tables S5–S19.

### 3.4. Maternal Outcomes

**Figure 2:**
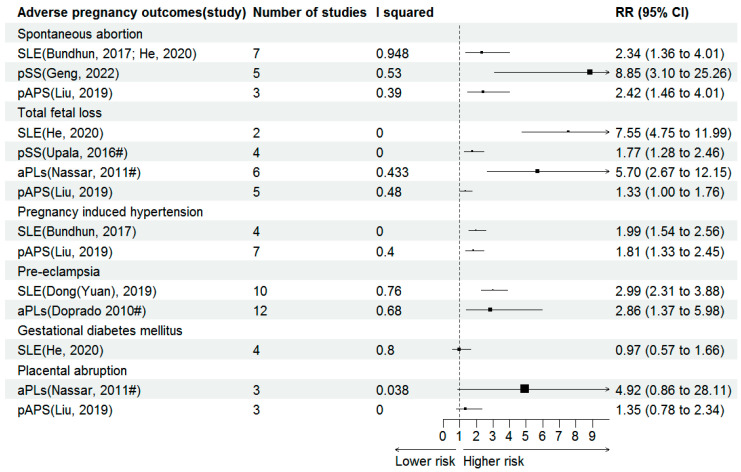
Forest plots for association of autoimmune conditions and maternal outcomes [27,33,35,36,40,42,43,44]. SLE, systemic lupus erythematosus; pSS, primary Sjögren syndrome; aPLs, antiphospholipid antibodies; pAPS, primary antiphospholipid syndrome. # Summary estimates are presented as odds ratios (ORs).

The forest plots of effect sizes from the included meta-analyses for maternal outcomes are shown in Figure 2.

Spontaneous Abortion

SLE, pSS and pAPS are all reported to be associated with an elevated risk of spontaneous abortion. In patients with SLE, the probability of spontaneous abortion is 2.34 times compared to the control group (RR 2.34, 95% CI 1.36–4.01). Higher risk of spontaneous abortion was identified in women with pSS (RR 8.85, 95% CI 3.10–25.26). We also observed that pAPS significantly increased the risk of spontaneous abortion (RR 2.42, 95% CI 1.46–4.01).

Total fetal loss

SLE and pAPS significantly increased the risk of total fetal loss according to the integrated results, with RR of 7.55 and 1.33, and 95% CI of 4.75–11.99 and 1.00–1.76 respectively. Moreover, pSS and positive aPLs were also associated with increased risk of total fetal loss, with OR of 1.77 and 5.70, 95% CI of 1.28–2.46 and 2.67–12.15, respectively.

PIH

Women with SLE had almost twofold higher risk of PIH (RR 1.99, 95% CI 1.54–2.56). The risk was also reported as near twofold higher in women with pAPS (RR 1.81, 95% CI 1.33–2.45).

PE

The risk of developing PE was almost threefold higher in women with SLE (RR 2.99, 95% CI 2.31–3.88). The odds of developing PE were nearly 3 times greater in women with positive aPLs (OR 2.86, 95% CI 1.37–5.98).

GDM

Only one meta-analysis explored the effects of SLE on the risk of GDM, but found that the risk of GDM was similar in women with or without SLE (RR 0.97, 95% CI 0.57–1.66).

PA

The association between positive aPLs and risk of PA was not significant (OR 4.92, 95% CI 0.86–28.11). Only one meta-analysis investigated the effects of pAPS on PA, but got negative result (RR 1.35, 95% CI 0.78–2.34).

### 3.5. Foetal/Neonatal Outcomes

**Figure 3:**
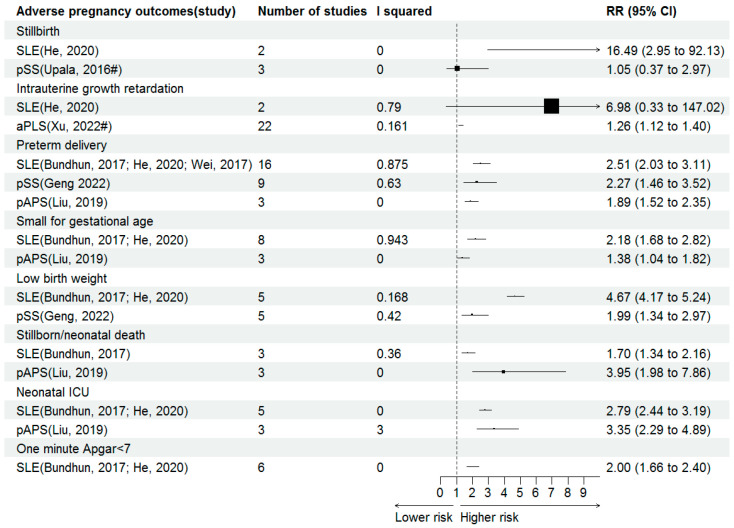
Forest plots for association of autoimmune conditions and fetal outcomes [26,33,35,37,40,42,44]. SLE, systemic lupus erythematosus; pSS, primary Sjögren syndrome; aPLs, antiphospholipid antibodies; pAPS, primary antiphospholipid syndrome. # Summary estimates are presented as odds ratios (ORs).

The forest plots of effect sizes from the included meta-analyses for foetal/neonatal outcomes are shown in Figure 3.

Stillbirth

The risk of developing stillbirth was almost sixteenfold higher in women with SLE (RR 16.49, 95% CI 2.95–92.13). The odds of developing stillbirth were similar in women with or without pSS (OR 1.05, 95% CI 0.37–2.97).

IUGR

No significant association was reported for IUGR in women with SLE (RR 6.98, 95% CI 0.33–147.02). Positive aPLs were reported to be associated with IUGR (OR 1.26, 95% CI 1.12–1.40).

Preterm delivery

Both SLE, pSS and pAPS were reported to increase the risk of premature delivery. The risk of developing premature delivery was nearly threefold in women with SLE than controls (RR 2.51, 95% CI 2.03–3.11). For the association between pSS and premature delivery, one meta-analysis reported the 2.27 times of risk in women with this disease (RR 2.27, 95% CI 1.46–3.52). The risk of premature delivery was nearly twofold higher in women with pAPS (RR 1.89, 95% CI 1.52–2.35).

SGA

Both SLE and pAPS were reported to increase the risk of SGA. Patients with SLE have a risk of SGA that is approximately 2.5 times higher than that of controls (RR 2.18, 95% CI 1.68–2.82). Women with pAPS exhibit an increased risk of SGA compared to those with negative (RR 1.38, 95% CI 1.04–1.82).

LBW

The risk of LBW in SLE patients exceeds that of controls by over approximately fivefold (RR 4.67, 95% CI 4.17–5.24). Patients with pSS exhibit approximately double the risk of LBW compared to those without (RR 1.99, 95% CI 1.34–2.97).

Stillborn/neonatal death

The risk of developing stillborn/neonatal death was almost twofold higher in women with SLE (RR 1.70, 95% CI 1.34–2.16). The risk of developing stillborn/neonatal death were also significantly incrased in women with pAPS (RR 3.95, 95% CI 1.98–7.86).

NICU

Neonatal admission to the NICU indicates a poorer pregnancy outcome. Therefore, the impact of SLE and pAPS on the NICU deserves attention. Both SLE and pAPS were reported to increase the risk of NICU, with RR of 2.79 and 3.35, 95% CI of 2.44–3.19 and 2.29–4.89.

One minute Apgar < 7

SLE was reported to increase the risk of fetal one-minute Apgar < 7 (RR 2.00, 95% CI 1.66–2.40).

### 3.6. Assisted Reproductive Outcomes

**Figure 4:**
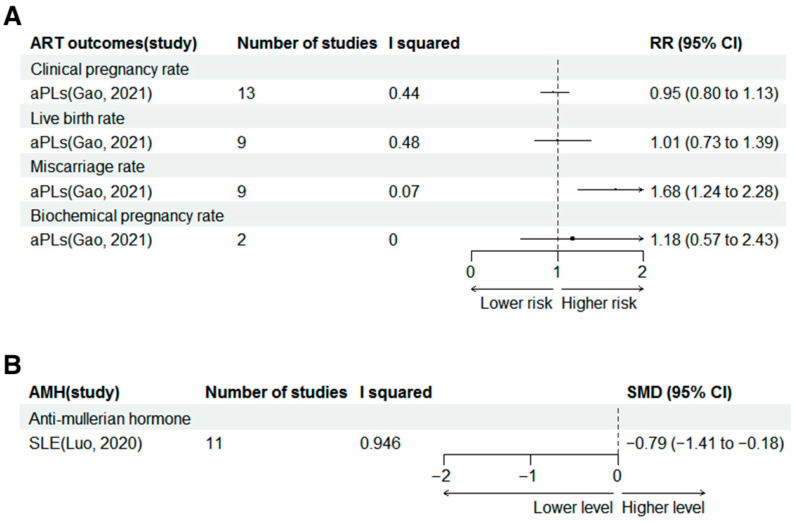
Forest plots for association of autoimmune conditions and assisted reproductive outcomes (**A**) [39] and AMH levels (**B**) [34]. SLE, systemic lupus erythematosus; pSS, primary Sjögren syndrome; aPLs, antiphospholipid antibodies; pAPS, primary antiphospholipid syndrome; ART, assisted reproductive technology; AMH, anti-mullerian hormone.

The forest plots of effect sizes from the included meta-analyses for assisted reproductive outcomes are shown in Figure 4.

Positive aPLs were reported to increase the risk of miscarriage rate in ART (RR 1.68, 95% CI 1.24–2.28). However, positive aPLs was not found to increase the risk of CPR, LBR and biochemical pregnancy rate in ART, with RR of 0.95, 1.01 and 1.18, 95% CI of 0.80–1.13, 0.73–1.39 and 0.57–2.43. These results are shown in Figure 4A.

### 3.7. AMH

Only a meta-analysis reported that SLE was related to the lower serum AMH level (SMD −0.79, 95% CI −1.4 to −0.18), as shown in Figure 4B.

## 4. Discussion

Women of childbearing age are particularly susceptible to autoimmune diseases, especially systemic autoimmune diseases such as SLE, SS and APS [45]. Managing these autoimmune diseases in women of childbearing age is challenging in clinical practice. Anti-rheumatic drugs must be used carefully to avoid gonadotoxicity and teratogenicity [46]. Pregnancy, whether through natural conception or ART, is a critical period for these patients with autoimmune diseases. It is essential to monitor disease activity to prevent disease flares and to focus on the effects of these autoimmune diseases on pregnancy and assisted reproductive outcomes [11,47]. However, the detailed phenotypes of pregnancy and assisted reproductive outcomes are very complex, and there is no consensus on the detailed influence of these autoimmune conditions on pregnancy and ART. Different influences often indicate different management strategies. Confirming the detailed effects of SLE, SS and APS on pregnancy and assisted reproductive outcomes based on the latest clinical evidence is crucial for guiding clinical practice.

It’s worth noting that the clinical diagnosis of these autoimmune disorders relies on specific manifestations and various autoantibodies [14,15,48]. For instance, positive anti-SSA/SSB antibodies are diagnostic markers for SS [15,16], while aPLs are diagnostic markers for APS [48,49]. However, these autoantibodies are not exclusive to their respective conditions; both anti-SSA/SSB antibodies and aPLs are also frequently found in SLE patients [13]. Anti-SSA/SSB antibodies and aPLs are recognized as pathogenic antibodies. Anti-SSA/SSB antibodies can target fetal heart cells to induce congenital heart block (CHB) [17,18], and aPLs can target trophoblasts and endothelial cells to induce trophoblast dysfunction and thrombosis [50]. Therefore, the effects of these autoantibodies in different clinical conditions do not depend on the diagnosis of autoimmune diseases. As such, in addition to SS and APS, this umbrella review was also designed to examine the effects of anti-SSA/SSB antibodies and aPLs on pregnancy and assisted reproductive outcomes.

**Figure 5:**
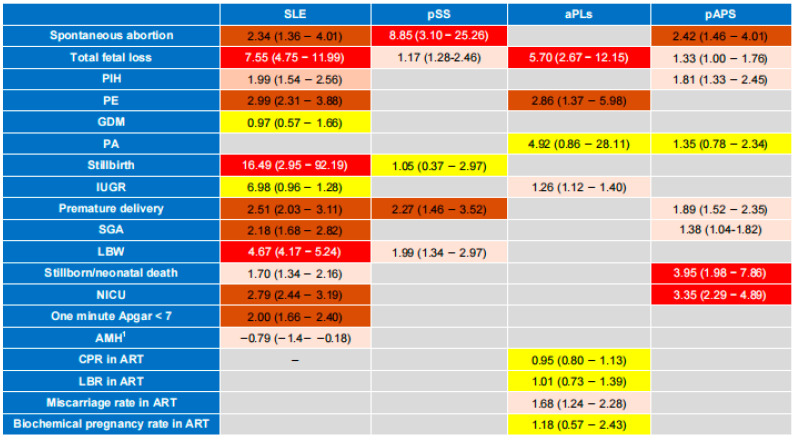
Heatmap showing the association of autoimmune conditions and pregnancy and assisted reproductive outcomes. ^1^ Quantitative outcomes, with effect sizes expressed using standardized mean difference. Red indicates an extremely significant impact (risk ratio or relative risk ≥ 3); brown indicates a very significant impact (2 ≤ risk ratio or relative risk < 3); pink indicates a significant impact (1 < risk ratio or relative risk < 2); yellow indicates a non-significant impact. SLE, systemic lupus erythematosus; pSS, primary Sjögren syndrome; aPLs, antiphospholipid antibodies; pAPS, primary antiphospholipid syndrome; AMH, anti-mullerian hormone; PIH, pregnancy induced hypertension; PE, pre-eclampsia; SGA, small for gestational age; LBW, low birth weight; NICU, neonatal intensive care unit; GDM, gestational diabetes mellitus; IUGR, intrauterine growth retardation; PA, placental abruption; CPR, clinical pregnancy rate; LBR, live birth ratel; ART, assisted reproductive technology.

This umbrella review provides the highest level of evidence currently available for the associations between SLE, SS, APS, and pregnancy and assisted reproductive outcomes. SLE was found to increase the risk of spontaneous abortion, fetal loss, PIH, PE, stillbirth, premature delivery, SGA, LBW, neonatal death, NICU and neonatal one-minute Apgar < 7, and to decrease AMH levels. However, the effects of SLE on GDM and IUGR were not supported by the evidence. pSS was found to increase the risk of spontaneous abortion, fetal loss, premature delivery, and LBW, but the effects on other pregnancy and assisted reproductive outcomes have rarely been explored in previous meta-analyses. However, there was no meta-analysis on the influence of anti-SSA/SSB antibodies on pregnancy and assisted reproductive outcomes included in this study. As important pathogenic autoantibodies, positive aPLs were associated with an increased risk of fetal loss, PE and IUGR. During ART, positive aPLs also increased the risk of miscarriage, but they did not affect the CPR, LBR and biochemical pregnancy rate in ART. The effects of positive aPLs on other pregnancy and assisted reproductive outcomes were not reported in the included meta-analyses. pAPS, an important autoimmune disease, increased the risk of spontaneous abortion, fetal loss, PIH, premature delivery, SGA, neonatal death and NICU, but it did not affect the occurrence of PA. The overall results of this umbrella review is shown in Figure 5.

Previous studies have identified that SLE increases the risk of early miscarriage, PE, eclampsia, emergency cesarean section, and preterm delivery [11]. Active or flaring SLE, active nephritis and hypertension are risk factors for adverse pregnancy outcomes in SLE patients [11]. In this study, we also confirmed that the effects of SLE on pregnancy outcomes are broad, including spontaneous abortion in early pregnancy and PIH, PE, IUGR, and premature delivery in late pregnancy. It is worth mentioning that we found that the risks of SGA and LBW were significantly higher in women with SLE, indicating that we should focus more on the infant health of SLE patients. For patients with SLE, it is recommended to conduct a detailed disease assessment before pregnancy and closely monitor disease activity during pregnancy [11]. SLE was also shown to decrease AMH levels, indicating diminished ovarian function. According to previous studies, the adverse influence of SLE on ovarian function is caused by the use of drugs with gonadotoxicity and teratogenicity [14,34]. However, the influence of SLE on assisted reproductive outcomes has rarely been explored in previous meta-analyses. It is possible that SLE is a contraindication for ART previously, but stable SLE patients were approved for receiving ART treatment in rencent years [12]. The ART outcomes in patients with stable SLE are worth exploring.

In previous studies, the influence of SS on pregnancy was rarely explored, but positive anti-SSA/SSB antibodies were regarded as independent risk factors for CHB according to some case reports and observational studies with small sample sizes [17,18]. A single-arm meta-analysis reported that the prevalence of premature delivery, cesarean operation, CHB, CHB recurrence, and cutaneous neonatal lupus erythematosus were 25%, 50%, 7%, 12%, and 19%, respectively [20]. Thus, pregnant women with positive anti-SSA/SSB antibodies were recommended to be monitored by fetal ultrasonic cardiogram, regardless of whether they had SLE, SLE-like disorders, SS, systemic sclerosis, or rheumatoid arthritis [12]. In this study, we found that pSS also increased the risk of spontaneous abortion, premature delivery, and LBW, not just restricted to CHB, providing new insights into the clinical management of SS patients. For all SS patients, with or without anti-SSA/SSB antibodies, treatment such as HCQ and other immunomodulators for pregnancy safety should be employed throughout the entire pregnancy. It is important to note that anti-SSA/SSB antibodies are not exclusively found in SS or SLE. These autoantibodies can also be detected in non-rheumatological conditions, particularly in autoimmune liver diseases such as primary biliary cholangitis (PBC), where anti-SSA/Ro52 antibodies may be present in a significant proportion of patients [51]. This has important clinical implications: the mere presence of anti-SSA/SSB antibodies does not automatically translate into a diagnosis of SS or SLE, and may not carry the same pregnancy risk profile as in patients with established autoimmune diseases. Future studies investigating pregnancy outcomes in anti-SSA/SSB-positive women should carefully distinguish between those with clinical autoimmune diagnoses and those with isolated autoantibody positivity, to better understand the true risk attributable to these antibodies.

APS is an important autoimmune disease characterized by thrombosis and pathologic pregnancies. Recently, additional manifestations such as lesions in microvessels, cardiac valves and hematologic abnormalities have been included in the classification criteria for APS [48]. Obstetric manifestations, including three or more consecutive miscarriages, stillbirth, early-onset PE, early placental insufficiency and premature delivery, are also included in the classification criteria for APS [48]. However, there is controversy regarding other clinical manifestations such as PA, two or more consecutive miscarriages, recurrent implantation failure (RIF) in ART, late-onset PE and late placental insufficiency. Whether these non-criteria manifestations should be included in the classification criteria for APS remains unresolved due to limited evidence of the clinical relationship between aPLs and these manifestations [52]. aPLs are a group of autoantibodies that target phospholipids or phospholipid-binding proteins. Lupus anticoagulant (LA), anticardiolipin antibody (aCL), and anti-β2 glycoprotein I (anti-β2GPI) are the most important aPLs included in the classification criteria for APS [48,49]. In vitro and in vivo studies have confirmed the pathogenic roles and mechanisms of aPLs in endothelial cells, trophoblasts and other cells such as endometrial stromal cells, monocytes and platelets [21,22]. Therefore, women with positive aPLs are recommended to be managed throughout the entire pregnancy with low-dose aspirin (LDA), low-molecular-weight heparin (LMWH), or a combination of both, regardless of the diagnosis of APS. When interpreting the association between aPLs and pregnancy outcomes, it is crucial to consider the heterogeneity introduced by differences in laboratory assays and the evolution of diagnostic criteria over time. The classification criteria for APS have undergone several revisions (e.g., the Sydney criteria in 2006), and variations in aPL detection methods—including different platforms, cut-off values, and isotype specificities—may contribute to inconsistencies across studies [53]. These methodological differences could partly explain the variability in reported associations between aPLs and specific obstetric manifestations. Future research should adopt standardized laboratory protocols and clearly define aPL positivity according to the latest consensus criteria to improve comparability across studies and enhance the quality of evidence for clinical decision-making. In this umbrella review, we found that positive aPLs increased the risk of PE and IUGR. aPLs were also shown to increase the miscarriage rate in ART but did not affect the CPR, LBR or biochemical pregnancy rate in ART. The effects of pAPS on spontaneous abortion, PIH, premature delivery and SGA were confirmed in this study, but pAPS did not increase the risk of PA according to the current clinical evidence. Based on these results, we suggest that RIF in ART and PA should not be included in the classification criteria for APS. However, pregnant women with positive aPLs or diagnosed APS should be closely monitored for the risks of spontaneous abortion, PIH, PE, IUGR, premature delivery, and SGA. For women with positive aPLs undergoing ART treatment, although the risk of RIF was not observed, LDA and/or LMWH should still be used to mitigate risks in subsequent pregnancies.

This umbrella review has several limitations. First, the number of original meta-analyses included in this study is still limited. Many associations between various autoimmune conditions and pregnancy and assisted reproductive outcomes have not been previously reported. For example, there is no evidence for the influence of SLE on CPR, LBR, miscarriage rate, and biochemical pregnancy rate in ART. The influence of pSS on assisted reproductive outcomes has also not been mentioned. The confusion in the management of assisted reproductive outcomes in women with these conditions remains unresolved. Second, the heterogeneity of the included meta-analyses cannot be removed under this study. Autoimmune conditions are complicated and heterogeneous, and different subtypes may determine different prognoses. The detailed aPLs and pAPS subtypes were not distinguished in this study, so we cannot obtain detailed information on the associations between specific aPLs (e.g., LA, aCL, and anti-β2GPI) and various pregnancy and assisted reproductive outcomes, which reduces the accuracy of the results. Third, this umbrella review focused specifically on SLE, SS, and APS. Other autoimmune conditions that may affect pregnancy outcomes, such as systemic sclerosis, were not included. Future umbrella reviews should consider expanding the scope to include these conditions as the evidence base grows.” Fourth, this umbrella review was unable to analyze the impact of potential confounders or effect modifiers, such as immunosuppressive treatment (including medication type, duration, and dosage), on pregnancy and assisted reproductive outcomes. The included meta-analyses rarely performed subgroup analyses based on treatment regimens, and primary studies often lacked detailed reporting of medication use. Lastly, the quality of the included original studies is relatively unsatisfactory in this field. In the future, more prospective studies with larger sample sizes and well-designed methodologies, as well as systematic reviews and meta-analyses based on them, may provide insights into the associations between SLE, SS, APS and complicated outcomes, ultimately benefiting clinical practice.

## 5. Conclusions

This review has provided a comprehensive summary of the current evidence regarding the associations between SLE, pSS, aPLs, pAPS and pregnancy and assisted reproductive outcomes. It also highlights the gaps and suggests future research directions in this field. Exploring the detailed influence of these autoimmune conditions on various pregnancy and assisted reproductive outcomes will benefit the optimization of clinical management guidelines for women with these conditions.

## Figures and Tables

**Table 1:** Basic information of included articles.

Author, Year	Deadline for Literature Retrieval	Number of Included Original Studies	Original Studies’ Type	Autoimmune Conditions	Outcomes	Quality Assessment Tool
Luo, 2020 [34]	April 2019	11	11 cross-sectional	SLE	AMH	None.
Bundhun, 2017 [35]	November 2016	11	1 cross-sectional 7 cohort 3 case-control	SLE	Spontaneous abortion, PIH, PE, Premature delivery, SGA, LBW, Stillborn/neonatal death, NICU, One minute Apgar < 7	None.
Wei, 2017 [37]	May 2016	6	6 case-control	SLE	Premature delivery	None.
Dong (Dai), 2019 [41]	August 2018	5	3 cohort and 2 case-control	SLE	GDM	NOS scale
He, 2020 [42]	December 2019	6	6 cohort	SLE	Spontaneous abortion, Total fetal loss, PE, GDM, Stillbirth, IUGR, Preterm delivery, SGA, LBW, NICU, One minute Apgar < 7	NOS scale
Dong (Yuan), 2019 [43]	June 2018	10	7 cohort 3 case-control	SLE	PE	NOS scale
Upala, 2016 [33]	March 2016	7	1 cross-sectional 4 cohort 2 case-control	pSS	Total fetal loss, Stillbirth, Premature delivery,	NOS scale
Geng, 2022 [44]	December 2021	9	7 cohort 2 case-control	pSS	Spontaneous abortion, Preterm delivery, LBW	NOS scale
do Prado, 2010 [36]	June 2009	12	8 cohort 4 case-control	aPLs	PE	Modified Downs and Black Checklist
Tan, 2022 [38]	April 2021	6	6 cohort	aPLs	CPR in ART, LBR in ART, Miscarriage rate in ART	NOS scale
Gao, 2021 [39]	February 2021	23	13 cohort 10 case-control	aPLs	CRP in ART, LBR in ART, Miscarriage rate in ART, Biochemical pregnancy rate in ART	NOS scale
Abou-Nassar, 2011 [27]	December 2009	28	8 cohort 20 case-control	aPLs	Total fetal loss, PE, PA, IUGR	NOS scale
Xu, 2022 [26]	November 2021	22	16 cohort 6 case-control	aPLs	IUGR	NOS scale
Liu, 2019 [40]	December 2015	8	5 cohort 3 case-control	pAPS	Spontaneous abortion, Total fetal loss, PIH, PA, Preterm delivery, SGA, Stillborn/neonatal death, NICU	NOS scale

Footnote: SLE, systemic lupus erythematosus; pSS, primary Sjögren syndrome; aPLs, antiphospholipid antibodies; pAPS, primary antiphospholipid syndrome; AMH, anti-mullerian hormone; PIH, pregnancy induced hypertension; PE, pre-eclampsia; SGA, small for gestational age; LBW, low birth weight; NICU, neonatal intensive care unit; GDM, gestational diabetes mellitus; IUGR, intrauterine growth retardation; PA, placental abruption; CPR, clinical pregnancy rate; LBR, live birth ratel; ART, assisted reproductive technology.

**Table 2:** Characteristics of included meta-analyses.

Author, Year	Exposures	Outcomes	Number of Included Original Studies	Sample Size (Cases/Participants)	*p* Value	Outlier Original Studies	Sensitivity Analysis (Yes/None)	*I* ^2^	Publication Bias	Results of Publication Bias	Evidence
Luo, 2020 [34]	SLE	AMH	11	535/937	0	One −0.21 (−0.40, −0.02)	Yes.	94.6	Funnel plot and Begg’s test	No significant	IV
Bundhun, 2017 [35]	SLE	Spontaneous abortion	4	420/2974	<0.0001	None.	Yes.	38	Funnel plot and Begg’s test	No significant	III
Bundhun, 2017 [35]	SLE	PIH	4	25,344/498,259	<0.00001	None.	Yes.	0	Funnel plot and Begg’s test	No significant	III
Bundhun, 2017 [35]	SLE	PE	4	14,984/288,306	<0.00001	None.	Yes.	44	Funnel plot and Begg’s test	No significant	III
Bundhun, 2017 [35]	SLE	Premature delivery	6	20,335/301,593	<0.00001	None.	Yes.	47	Funnel plot and Begg’s test	No significant	III
Bundhun, 2017 [35]	SLE	SGA	4	57,063/475,404	<0.00001	None.	Yes.	0	Funnel plot and Begg’s test	No significant	III
Bundhun, 2017 [35]	SLE	LBW	2	12,652/270,467	<0.00001	None.	Yes.	0	Funnel plot and Begg’s test	No significant	III
Bundhun, 2017 [35]	SLE	Stillborn/neonatal death	3	123/2532	<0.00001	None.	Yes.	36	Funnel plot and Begg’s test	No significant	III
Bundhun, 2017 [35]	SLE	NICU	3	48,087/469,344	<0.00001	None.	Yes.	0	Funnel plot and Begg’s test	No significant	III
Bundhun, 2017 [35]	SLE	One minute Apgar < 7	3	42,320/481,919	<0.00001	None.	Yes.	0	Funnel plot and Begg’s test	No significant	III
Wei, 2017 [37]	SLE	Premature delivery	6	1545/3690	0.01	None.	None.	66.5	Begg’s test and funnel plot	No significant	IV
Dong (Dai), 2019 [41]	SLE	GDM	5	248/3432	0.848	None.	Yes.	76	None.	-	NS
He, 2020 [42]	SLE	Spontaneous abortion	3	100,433/8,792,890	<0.00001	None.	Yes.	66	Funnel plots	No significant	III
He, 2020 [42]	SLE	Total fetal loss	2	87/2414	<0.00001	None.	Yes.	0	Funnel plots	No significant	III
He, 2020 [42]	SLE	PE	4	402,412/879,4417	<0.00001	None.	Yes.	32	Funnel plots	No significant	III
He, 2020 [42]	SLE	GDM	4	1494/13,993	0.92	None.	Yes.	80	Funnel plots	No significant	NS
He, 2020 [42]	SLE	Stillbirth	2	11/1499	0.001	None.	Yes.	0	Funnel plots	No significant	III
He, 2020 [42]	SLE	IUGR	2	106/1448	0.21	None.	Yes.	79	Funnel plots	No significant	NS
He, 2020 [42]	SLE	Premature delivery	6	711,193/8,805,962	<0.00001	None.	Yes.	94	Funnel plots	No significant	III
He, 2020 [42]	SLE	SGA	4	2237/20,616	0.002	None.	Yes.	97	Funnel plots	No significant	IV
He, 2020 [42]	SLE	LBW	3	758/8501	<0.00001	None.	Yes.	59	Funnel plots	No significant	III
He, 2020 [42]	SLE	NICU	2	390/4917	<0.00001	None.	Yes.	0	Funnel plots	No significant	III
He, 2020 [42]	SLE	One minute Apgar < 7	3	142/2975	<0.00001	None.	Yes.	0	Funnel plots	No significant	III
Dong (Yuan), 2019 [43]	SLE	PE	10	426,914/9,462,549	<0.001	None.	Yes.	76	Funnel plots, Egger’s linear regression and Begg’s rank correlation test	No significant	III
Upala, 2016 [33]	pSS	Total fetal loss	4	602/1537	0.01	None.	None.	0	None	-	IV
Upala, 2016 [33]	pSS	Stillbirth	3	292/1951	0.92	None.	None.	0	None.	-	NS
Upala, 2016 [33]	pSS	Premature delivery	5	342/2217	0.25	None.	None.	59	None.	-	NS
Geng, 2022 [44]	pSS	Spontanrous abortion	5	42/1284	0.071	None.	Yes.	53	Begg’s test with Egger’s test	Significant	NS
Geng, 2022 [44]	pSS	Premature delivery	9	245/14,515,204	0.006	None.	Yes.	63	Begg’s test with Egger’s test	No significant	IV
Geng, 2022 [44]	pSS	LBW	5	23/959	0.142	None.	Yes.	42	Begg’s test with Egger’s test	No significant	NS
do Prado, 2010 [36]	aPLs	PE	12	1636/5704	<0.001	One 2.86 (1.37, 5.98)	None.	68.7	Funnel plot and Egger test	No significant	III
Tan, 2022 [38]	aPLs	CPR in ART	6	1684/3214	0.42	None.	None.	32	Egger test	No significant	NS
Tan, 2022 [38]	aPLs	LBR in ART	5	1666/2943	0.52	None.	None.	61	Egger test	No significant	NS
Tan, 2022 [38]	aPLs	Miascrriage rate in ART	6	214/1685	0.14	None.	None.	0	Egger test	No significant	NS
Gao, 2021 [39]	aPLs	CPR in ART	13	1741/3954	0.57	None.	Yes.	44	Funnel plots	No significant	NS
Gao, 2021 [39]	aPLs	LBR in ART	9	987/3403	0.96	None.	Yes.	48	Funnel plots	No significant	NS
Gao, 2021 [39]	aPLs	Miascrriage rate in ART	9	236/1702	0.0007	None.	Yes.	7	Funnel plots	No significant	III
Gao, 2021 [39]	aPLs	Biochemical pregnancy rate in ART	2	35/469	0.65	None.	Yes.	0	Funnel plots	No significant	NS
Abou-Nassar, 2011 [27]	aPLs	Total fetal loss	6	130/2798	/	None.	Yes.	43.3	None.	-	NS
Abou-Nassar, 2011 [27]	aPLs	PE	23	10/5981	/	None.	Yes.	0	None.	-	NS
Abou-Nassar, 2011 [27]	aPLs	PA	3	32/1044	/	None.	Yes.	3.8	None.	-	NS
Abou-Nassar, 2011 [27]	aPLs	IUGR	7	3/3152	/	None.	Yes.	0	None.	-	NS
Xu, 2022 [26]	aPLs	IUGR	22	1188/11,745	0.245	None.	Yes.	16.1	Funnel plots and Begg’s test	No significant	NS
Liu, 2019 [40]	pAPS	Spontaneous abortion	3	66/396	0.0006	None.	Yes.	39	Funnel plots	No significant	IV
Liu, 2019 [40]	pAPS	Total fetal loss	5	170/1758	0.05	None.	Yes.	48	Funnel plots	No significant	NS
Liu, 2019 [40]	pAPS	PIH	7	20,593/212,887	0.0002	None.	Yes.	40	Funnel plots	No significant	III
Liu, 2019 [40]	pAPS	PA	3	46/1532	0.29	None.	Yes.	0	Funnel plots	No significant	NS
Liu, 2019 [40]	pAPS	Premature delivery	3	278/1520	<0.00001	None.	Yes.	0	Funnel plots	No significant	III
Liu, 2019 [40]	pAPS	SGA	3	175/1532	0.02	None.	Yes.	0	Funnel plots	No significant	IV
Liu, 2019 [40]	pAPS	Stillborn/neonatal death	3	41/1551	<0.0001	None.	Yes.	0	Funnel plots	No significant	III
Liu, 2019 [40]	pAPS	NICU	3	22,859/211,264	<0.00001	None.	Yes.	3	Funnel plots	No significant	III

Footnote: Abbreviations as in Table 1.

**Table 3:** The results of overlapping analyses in this umbrella review.

	SLE	pSS	aPLs	pAPS
Spontaneous abortion	Bundhun, 2017 [35]; He, 2020 [42] CCA 0%	Geng, 2022 [44]		Liu, 2019 [40]
Total fetal loss	He, 2020 [42]	Upala, 2016 [33]	Abou-Nassar, 2011 [27]	Liu, 2019 [40]
PIH	Bundhun, 2017 [35]			Liu, 2019 [40]
PE	Bundhun, 2017 [35]; He, 2020 [42]; Dong (Yuan), 2019 [43] CCA 30.76%		Abou-Nassar, 2011 [27]; do Prado, 2010 [36] CCA 26.92%	
GDM	Dong (Dai), 2019 [41]; He, 2020 [42] CCA 50%			
PA			Abou-Nassar, 2011 [27]	Liu, 2019 [40]
Stillbirth	He, 2020 [42]	Upala, 2016 [33]		
IUGR	He, 2020 [42]		Abou-Nassar, 2011 [27]; Xu, 2022 [26] CCA 20.83%	
Premature delivery	Bundhun, 2017 [35]; Wei, 2017 [37]; He, 2020 [42] CCA 6.25%	Upala, 2016 [33]; Geng, 2022 [44] CCA 16.67%		Liu, 2019 [40]
SGA	Bundhun, 2017 [35]; He, 2020 [42] CCA 0%			Liu, 2019 [40]
LBW	Bundhun, 2017 [35]; He, 2020 [42] CCA 0%	Geng, 2022 [44]		
Stillborn/neonatal death	Bundhun, 2017 [35]			Liu, 2019 [40]
NICU	Bundhun, 2017 [35]; He, 2020 [42] CCA 0%			Liu, 2019 [40]
One minute Apgar < 7	Bundhun, 2017 [35]; He, 2020 [42] CCA 0%			
AMH	Luo, 2020 [34]			
CPR in ART			Tan, 2022 [38]; Gao, 2021 [39] CCA 35.71%	
LBR in ART			Tan, 2022 [38]; Gao, 2021 [39] CCA 40%	
Miscarriage rate in ART			Tan, 2022 [38]; Gao, 2021 [39] CCA 50%	
Biochemical pregnancy rate in ART			Gao, 2021 [39]	

Footnote: Abbreviations as in Table 1.

## Data Availability

All data generated and analyzed during this study are included in this article.

## Supplementary Materials

The following supporting information can be downloaded at: https://www.mdpi.com/article/10.3390/jcm15072618/s1, Figure S1. The meta-analysis on the association between SLE and spontaneous abortion; Figure S2. Funnel plot for meta-analysis on the association between SLE and spontaneous abortion; Figure S3. The meta-analysis on the association between SLE and premature delivery; Figure S4. Funnel plot for meta-analysis on the association between SLE and premature delivery; Figure S5. The meta-analysis on the association between SLE and SGA; Figure S6. Funnel plot for meta-analysis on the association between SLE and SGA; Figure S7. The meta-analysis on the association between SLE and LBW; Figure S8. Funnel plot for meta-analysis on the association between SLE and LBW; Figure S9. The meta-analysis on the association between SLE and NICU; Figure S10. Funnel plot for meta-analysis on the association between SLE and NICU; Figure S11. The meta-analysis on the association between SLE and one minute Apgar < 7; Figure S12. Funnel plot for meta-analysis on the association between SLE and one minute Apgar < 7. Table S1. Search strategy in the Pubmed database; Table S2. Search strategy in the Embase (Ovid) database; Table S3. Search strategy in the Cochrane database of systematic reviews; Table S4. Quality assessment of included articles using AMSTAR 2 tool; Table S5. Overlapping between articles for the association between SLE and spontaneous abortion; Table S6. Overlapping between articles for the association between SLE and PE; Table S7. Overlapping between articles for the association between SLE and GDM; Table S8. Overlapping between articles for the association between SLE and premature delivery; Table S9. Overlapping between articles for the association between SLE and SGA; Table S10. Overlapping between articles for the association between SLE and LBW; Table S11. Overlapping between articles for the association between SLE and NICU; Table S12. Overlapping between articles for the association between SLE and One minute Apgar < 7; Table S13. Overlapping between articles for the association between pSS and premature delivery; Table S14. Overlapping between articles for the association between aPLs and PE; Table S15. Overlapping between articles for the association between aPLs and IUGR; Table S16. Overlapping between articles for the association between aPLs and CPR in ART; Table S17. Overlapping between articles for the association between aPLs and LBR in ART; Table S18. Overlapping between articles for the association between aPLs and Miscarriage rate in ART; Table S19. Overlapping and non overlapping associations of included meta-analyses by CCA calculations; Table S20. The results of meta-analysis. Supplementary Material S1. PRISMA 2020 Checklist [54].
