# Supplementary material for: Pregnancy and Assisted Reproductive Outcomes in Women with Systemic Lupus Erythematosus, Sjögren Syndrome and Antiphospholipid Syndrome: An Umbrella Review
Source: J Clin Med. 2026 Mar 30;15(7):2618. doi: 10.3390/jcm15072618 (PMC13072902; doi:10.3390/jcm15072618)
Supplement: Supplementary file 1 [file jcm-15-02618-s001.zip › Supplementary Figrues.pdf]

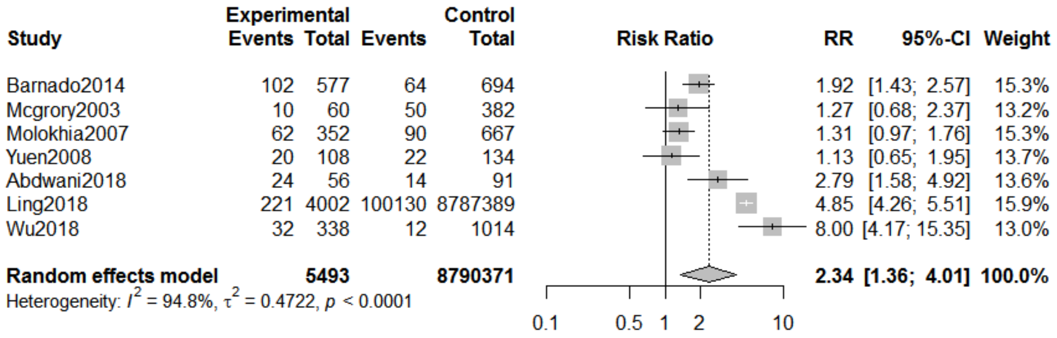

Figure S1. The meta-analysis on the association between SLE and spontaneous abortion

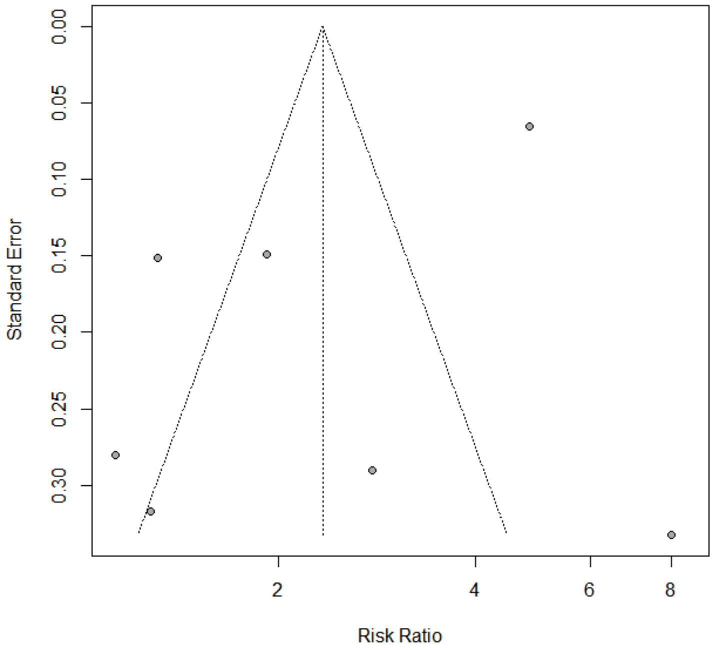

Figure S2. Funnel plot for meta-analysis on the association between SLE and spontaneous abortion

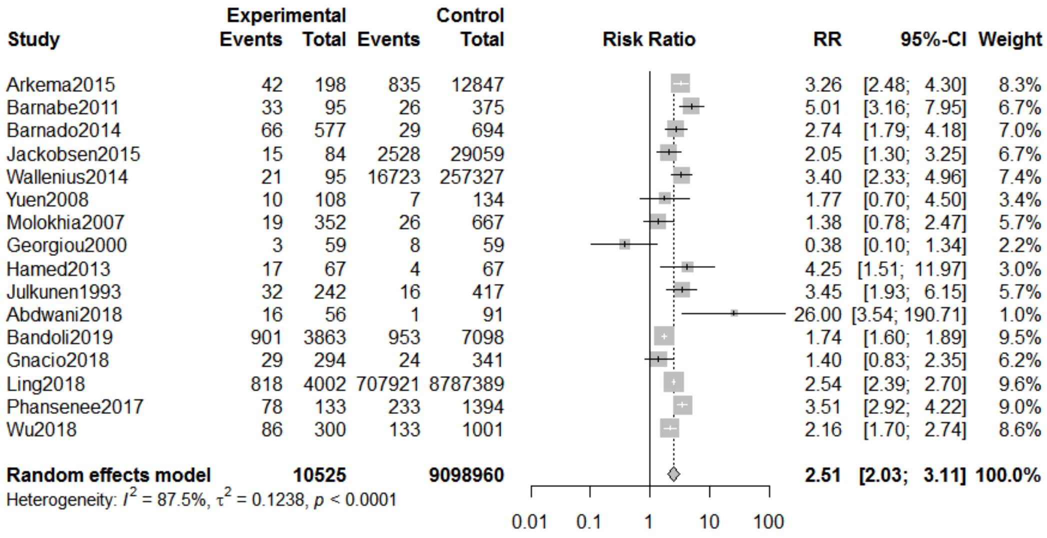

Figure S3. The meta-analysis on the association between SLE and premature delivery

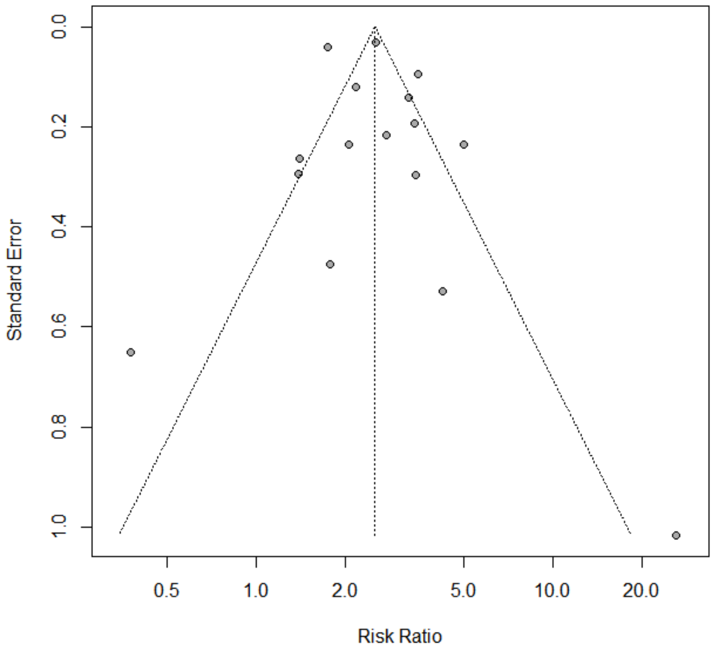

Figure S4. Funnel plot for meta-analysis on the association between SLE and premature delivery

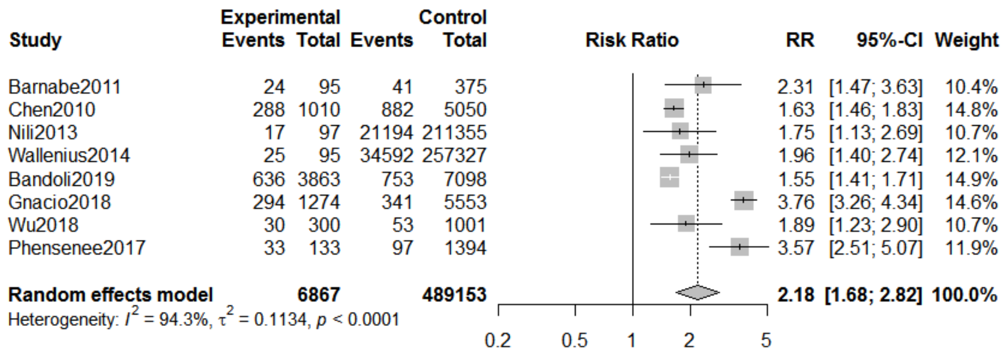

Figure S5. The meta-analysis on the association between SLE and SGA

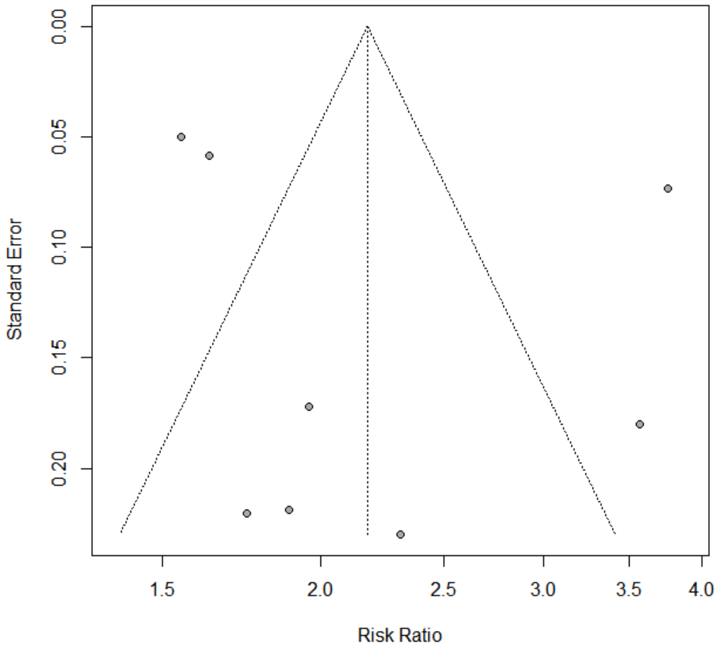

Figure S6. Funnel plot for meta-analysis on the association between SLE and SGA

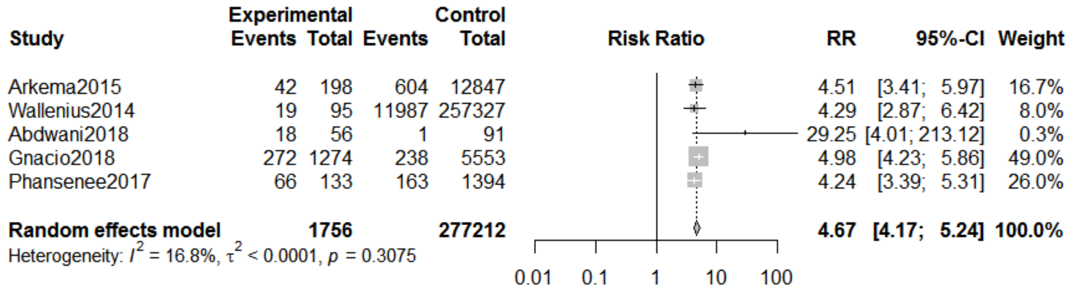

Figure S7. The meta-analysis on the association between SLE and LBW

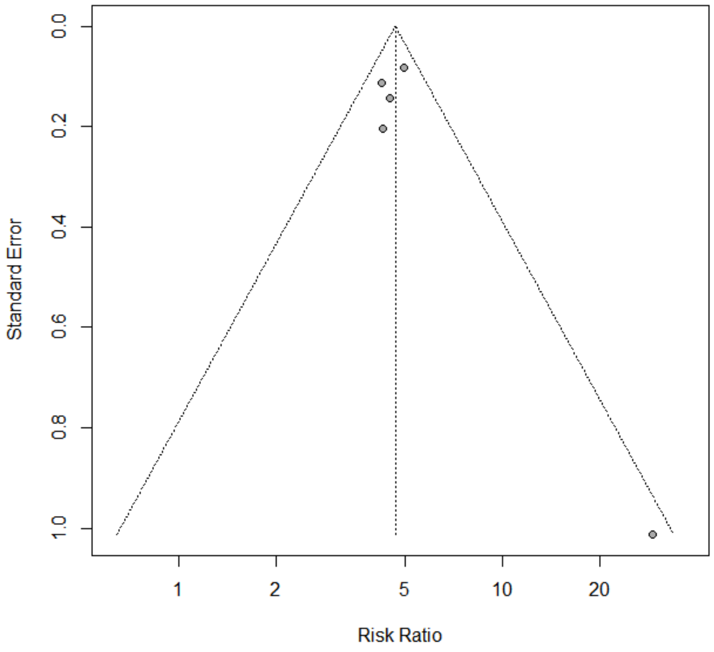

Figure S8. Funnel plot for meta-analysis on the association between SLE and LBW

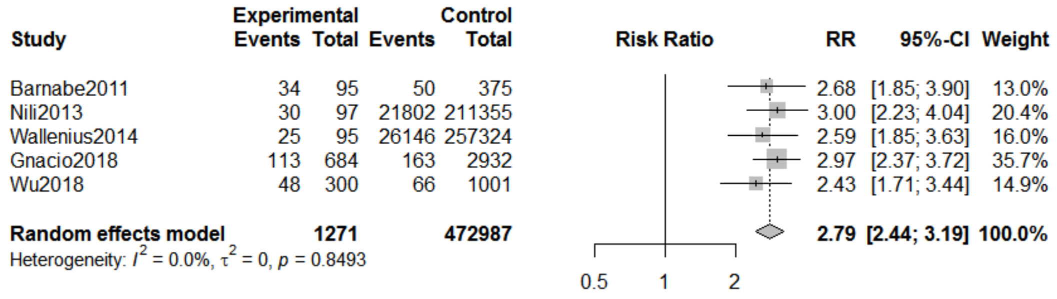

Figure S9. The meta-analysis on the association between SLE and NICU

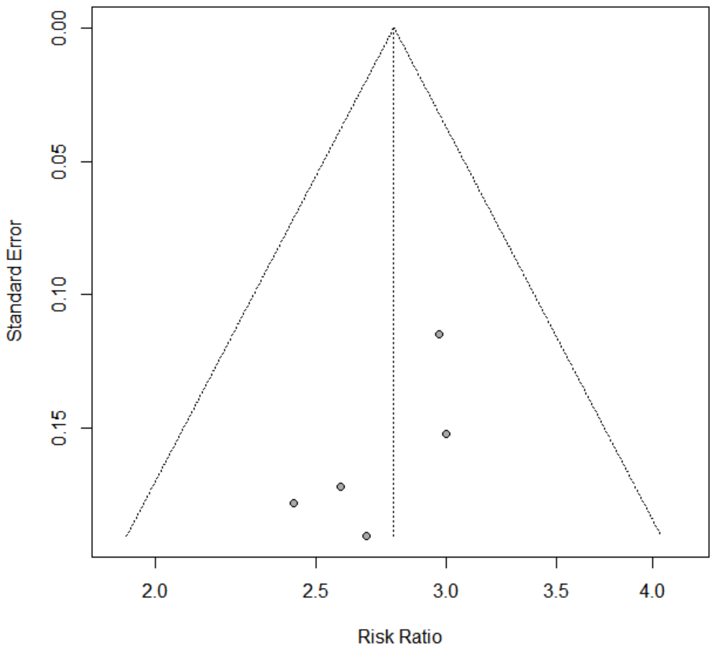

Figure S10. Funnel plot for meta-analysis on the association between SLE and NICU

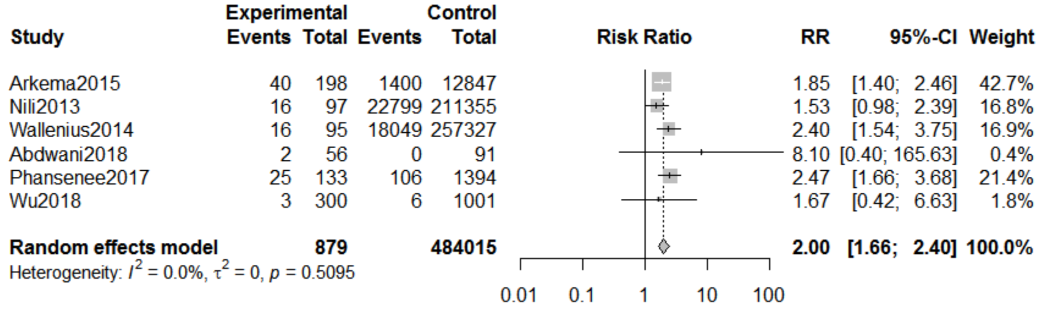

Figure S11. The meta-analysis on the association between SLE and one minute Apgar < 7

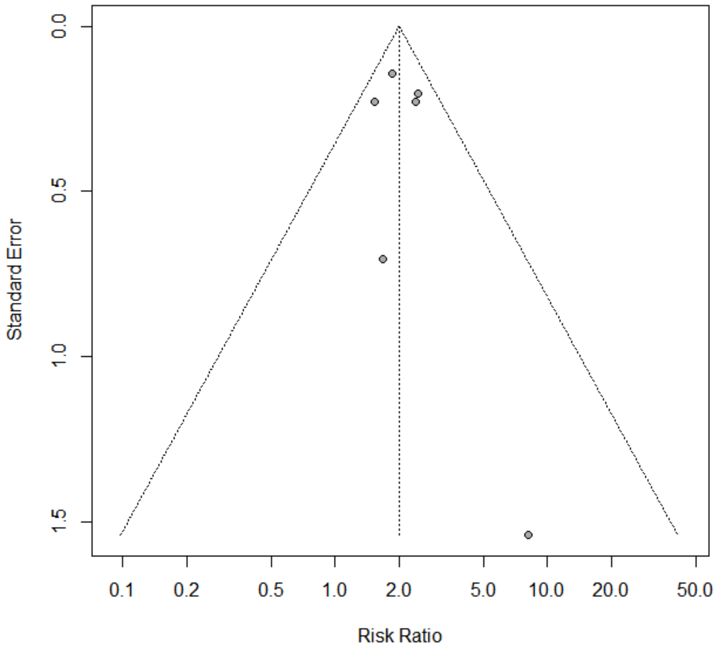

Figure S12. Funnel plot for meta-analysis on the association between SLE and one minute Apgar < 7
