# Supplementary material for: Pregnancy and Assisted Reproductive Outcomes in Women with Systemic Lupus Erythematosus, Sjögren Syndrome and Antiphospholipid Syndrome: An Umbrella Review
Source: J Clin Med. 2026 Mar 30;15(7):2618. doi: 10.3390/jcm15072618 (PMC13072902; doi:10.3390/jcm15072618)
Supplement: Supplementary file 1 [file jcm-15-02618-s001.zip › Supplementary Tables.pdf]

Supplementary Tables

Table S1. Search strategy in the Pubmed database.

Search number	Query
#1	"Antibodies, Antiphospholipid"[Mesh]
#2	((((((((Antiphospholipid Autoantibody[Title/Abstract]) OR (Autoantibody, Antiphospholipid[Title/Abstract])) OR (Phospholipid Autoantibody[Title/Abstract])) OR (Autoantibody, Phospholipid[Title/Abstract])) OR (Antiphospholipid Antibody[Title/Abstract])) OR (Antibody, Antiphospholipid[Title/Abstract])) OR (Phospholipid Autoantibodies[Title/Abstract])) OR (Antiphospholipid Autoantibodies[Title/Abstract])) OR (Antiphospholipid Antibodies[Title/Abstract])
#3	#1 OR #2
#4	"Antiphospholipid Syndrome"[Mesh]
#5	(((((((((((((Syndrome, Antiphospholipid[Title/Abstract]) OR (Hughes Syndrome[Title/Abstract])) OR (Syndrome, Hughes[Title/Abstract])) OR (Antiphospholipid Antibody Syndrome[Title/Abstract])) OR (Antibody Syndrome, Antiphospholipid[Title/Abstract])) OR (Antiphospholipid Antibody Syndromes[Title/Abstract])) OR (Syndrome, Antiphospholipid Antibody[Title/Abstract])) OR (Anti-Phospholipid Antibody Syndrome[Title/Abstract])) OR (Anti Phospholipid Antibody Syndrome[Title/Abstract])) OR (Antibody Syndrome, Anti-Phospholipid[Title/Abstract])) OR (Syndrome, Anti-Phospholipid Antibody[Title/Abstract])) OR (Anti-Phospholipid Syndrome[Title/Abstract])) OR (Anti Phospholipid Syndrome[Title/Abstract])) OR (Syndrome, Anti-Phospholipid[Title/Abstract])
#6	#4 OR #5
#7	"Lupus Erythematosus, Systemic"[Mesh]
#8	(((((Systemic Lupus Erythematosus[Title/Abstract]) OR (Lupus Erythematosus Disseminatus[Title/Abstract])) OR (Libman-Sacks Disease[Title/Abstract])) OR (Disease, Libman-Sacks[Title/Abstract])) OR (Libman Sacks Disease[Title/Abstract])) OR (systematic lupus erythematosus[Title/Abstract])
#9	#7 OR #8
#10	"SS-A antibodies" [Supplementary Concept]
#11	((((((((((Sjogren's Anti-SS-A antibodies[Title/Abstract]) OR (Sjogren's Anti-SS-A autoantibody[Title/Abstract])) OR (Sjogren's Anti-SS-A autoantibodies[Title/Abstract])) OR (SS A antibodies[Title/Abstract])) OR (Anti-Ro SSA autoantibodies[Title/Abstract])) OR (Anti-SSA autoantibodies[Title/Abstract])) OR (anti-Ro52 autoantibodies[Title/Abstract])) OR (anti-SSA Ro52 autoantibodies[Title/Abstract])) OR (Ro antibodies[Title/Abstract])) OR (Sjogren's Anti-SS-A antibody[Title/Abstract])) OR (Anti-Ro autoantibodies[Title/Abstract])
#12	#10 OR #11
#13	(((SS B antibod*[Title/Abstract]) OR (SSB autoantibod*[Title/Abstract])) OR (La antibod*[Title/Abstract])) OR (La

	autoantibod*[Title/Abstract])
#14	#3 OR #6 OR #9 OR #12 OR #13
#15	"Ovarian Function Tests"[Mesh]
#16	(((((((Function Test, Ovarian[Title/Abstract]) OR (Function Tests, Ovarian[Title/Abstract])) OR (Ovarian Function Test[Title/Abstract])) OR (Test, Ovarian Function[Title/Abstract])) OR (Tests, Ovarian Function[Title/Abstract])) OR (basal follicle- stimulating hormone[Title/Abstract])) OR (basal estrogen[Title/Abstract])) OR (antral follicle count[Title/Abstract])
#17	#15 OR #16
#18	"Ovarian Reserve"[Mesh]
#19	((Ovarian Reserves[Title/Abstract]) OR (Reserve, Ovarian[Title/Abstract])) OR (Reserves, Ovarian[Title/Abstract])
#20	#18 OR #19
#21	"Anti-Mullerian Hormone"[Mesh]
#22	(((((((((((((Anti Mullerian Hormone[Title/Abstract]) OR (Mullerian-Inhibiting Factor[Title/Abstract])) OR (Mullerian Inhibiting Factor[Title/Abstract])) OR (Anti-Mullerian Factor[Title/Abstract])) OR (Anti Mullerian Factor[Title/Abstract])) OR (Mullerian-Inhibitory Substance[Title/Abstract])) OR (Mullerian Inhibitory Substance[Title/Abstract])) OR (Mullerian Inhibiting Hormone[Title/Abstract])) OR (Mullerian Inhibiting Substance[Title/Abstract])) OR (Mullerian Regression Factor[Title/Abstract])) OR (Mullerian-Inhibiting Hormone[Title/Abstract])) OR (Anti-Muellerian Hormone[Title/Abstract])) OR (Anti Muellerian Hormone[Title/Abstract])) OR (Hormone, Anti-Muellerian[Title/Abstract])) OR (Antimullerian Hormone[Title/Abstract])
#23	#21 OR #22
#24	"Fertility"[Mesh]
#25	((((((((((((((((((((((((Fecundability[Title/Abstract]) OR (Fecundity[Title/Abstract])) OR (Differential Fertility[Title/Abstract])) OR (Fertility, Differential[Title/Abstract])) OR (Fertility Determinants[Title/Abstract])) OR (Determinant, Fertility[Title/Abstract])) OR (Determinants, Fertility[Title/Abstract])) OR (Fertility Determinant[Title/Abstract])) OR (Subfecundity[Title/Abstract])) OR (Fertility Preferences[Title/Abstract])) OR (Fertility Preference[Title/Abstract])) OR (Preference, Fertility[Title/Abstract])) OR (Preferences, Fertility[Title/Abstract])) OR (Fertility, Below Replacement[Title/Abstract])) OR (Below Replacement Fertility[Title/Abstract])) OR (Marital Fertility[Title/Abstract])) OR (Fertility, Marital[Title/Abstract])) OR (Natural Fertility[Title/Abstract])) OR (Fertility, Natural[Title/Abstract])) OR (World Fertility Survey[Title/Abstract])) OR (Fertility Survey, World[Title/Abstract])) OR (Fertility Surveys, World[Title/Abstract])) OR (Survey, World Fertility[Title/Abstract])) OR (Surveys, World Fertility[Title/Abstract])) OR (World Fertility Surveys[Title/Abstract])) OR (Fertility Incentives[Title/Abstract])) OR (Fertility Incentive[Title/Abstract])
#26	#24 OR #25
#27	"Infertility, Female"[Mesh]
#28	(((((((((Female Infertility[Title/Abstract]) OR (Sterility, Postpartum[Title/Abstract])) OR (Postpartum Sterility[Title/Abstract])) OR (Subfertility, Female[Title/Abstract])) OR (Female Subfertility[Title/Abstract])) OR (Sub-Fertility, Female[Title/Abstract])) OR (Female Sub-Fertility[Title/Abstract])) OR (Sub Fertility, Female[Title/Abstract])) OR (Sterility, Female[Title/Abstract])) OR (Female

	Sterility[Title/Abstract])
#29	#27 OR #28
#30	"Reproductive Techniques, Assisted"[Mesh]
#31	(((((((((((((((Assisted Reproductive Technique[Title/Abstract]) OR (Reproductive Technique, Assisted[Title/Abstract])) OR (Technique, Assisted Reproductive[Title/Abstract])) OR (Techniques, Assisted Reproductive[Title/Abstract])) OR (Assisted Reproductive Technics[Title/Abstract])) OR (Assisted Reproductive Technic[Title/Abstract])) OR (Reproductive Technic, Assisted[Title/Abstract])) OR (Reproductive Technics, Assisted[Title/Abstract])) OR (Technic, Assisted Reproductive[Title/Abstract])) OR (Technics, Assisted Reproductive[Title/Abstract])) OR (Assisted Reproductive Techniques[Title/Abstract])) OR (Reproductive Technology, Assisted[Title/Abstract])) OR (Assisted Reproductive Technologies[Title/Abstract])) OR (Assisted Reproductive Technology[Title/Abstract])) OR (Reproductive Technologies, Assisted[Title/Abstract])) OR (Technologies, Assisted Reproductive[Title/Abstract])) OR (Technology, Assisted Reproductive[Title/Abstract])
#32	#30 OR #31
#33	"Sperm Injections, Intracytoplasmic"[Mesh]
#34	((((((Injection, Intracytoplasmic Sperm[Title/Abstract]) OR (Injections, Intracytoplasmic Sperm[Title/Abstract])) OR (Intracytoplasmic Sperm Injection[Title/Abstract])) OR (Sperm Injection, Intracytoplasmic[Title/Abstract])) OR (Intracytoplasmic Sperm Injections[Title/Abstract])) OR (ICSI[Title/Abstract])) OR (Injections, Sperm, Intracytoplasmic[Title/Abstract])
#35	#33 OR #34
#36	"Fertilization in Vitro"[Mesh]
#37	(((((((((((In Vitro Fertilization[Title/Abstract]) OR (In Vitro Fertilizations[Title/Abstract])) OR (Test-Tube Fertilization[Title/Abstract])) OR (Fertilization, Test-Tube[Title/Abstract])) OR (Fertilizations, Test-Tube[Title/Abstract])) OR (Test Tube Fertilization[Title/Abstract])) OR (Test-Tube Fertilizations[Title/Abstract])) OR (Fertilizations in Vitro[Title/Abstract])) OR (Test-Tube Babies[Title/Abstract])) OR (Babies, Test-Tube[Title/Abstract])) OR (Baby, Test-Tube[Title/Abstract])) OR (Test Tube Babies[Title/Abstract])) OR (Test-Tube Baby[Title/Abstract])
#38	#36 OR #37
#39	"Pregnancy Outcome"[Mesh]
#40	((Pregnancy Outcomes[Title/Abstract]) OR (Outcome, Pregnancy[Title/Abstract])) OR (Outcomes, Pregnancy[Title/Abstract])
#41	(Clinical pregnancy rate[Title/Abstract]) OR (live birth rate[Title/Abstract])
#42	#39 OR #40 OR #41
#43	#32 OR #35 OR #38
#44	#42 OR #43
#45	#17 OR #20 OR #23OR #26OR #29
#46	"Pregnancy Complications"[Mesh]
#47	((((((Complication, Pregnancy[Title/Abstract]) OR (Pregnancy Complication[Title/Abstract])) OR (Complications, Pregnancy[Title/Abstract])) OR (Adverse Birth Outcomes[Title/Abstract])) OR (Adverse Birth Outcome[Title/Abstract])) OR (Birth Outcome, Adverse[Title/Abstract])) OR

	(Outcome, Adverse Birth[Title/Abstract])
#48	#46 OR #47
#49	"Abortion, Spontaneous"[Mesh]
#50	((((((((((((((Abortions, Spontaneous[Title/Abstract]) OR (Spontaneous Abortions[Title/Abstract])) OR (Spontaneous Abortion[Title/Abstract])) OR (Early Pregnancy Loss[Title/Abstract])) OR (Early Pregnancy Losses[Title/Abstract])) OR (Loss, Early Pregnancy[Title/Abstract])) OR (Losses, Early Pregnancy[Title/Abstract])) OR (Pregnancy Loss, Early[Title/Abstract])) OR (Pregnancy Losses, Early[Title/Abstract])) OR (Miscarriage[Title/Abstract])) OR (Miscarriages[Title/Abstract])) OR (Abortion, Tubal[Title/Abstract])) OR (Abortions, Tubal[Title/Abstract])) OR (Tubal Abortion[Title/Abstract])) OR (Tubal Abortions[Title/Abstract])
#51	#49 OR #50
#52	"Abortion, Induced"[Mesh]
#53	((Induced Abortion[Title/Abstract]) OR (Abortions, Induced[Title/Abstract])) OR (Induced Abortions[Title/Abstract])) OR (Abortion (Induced[Title/Abstract]))) OR (Abortions (Induced[Title/Abstract]))) OR (Abortion Rate[Title/Abstract])) OR (Abortion Rates[Title/Abstract])) OR (Rate, Abortion[Title/Abstract])) OR (Rates, Abortion[Title/Abstract])) OR (Abortion Techniques[Title/Abstract])) OR (Abortion Technique[Title/Abstract])) OR (Technique, Abortion[Title/Abstract])) OR (Techniques, Abortion[Title/Abstract])) OR (Abortion Technics[Title/Abstract])) OR (Abortion Technic[Title/Abstract])) OR (Technic, Abortion[Title/Abstract])) OR (Technics, Abortion[Title/Abstract])) OR (Abortion, Drug-Induced[Title/Abstract])) OR (Abortion, Drug Induced[Title/Abstract])) OR (Abortions, Drug-Induced[Title/Abstract])) OR (Drug-Induced Abortion[Title/Abstract])) OR (Drug-Induced Abortions[Title/Abstract])) OR (Previous Abortion[Title/Abstract])) OR (Abortion, Previous[Title/Abstract])) OR (Abortions, Previous[Title/Abstract])) OR (Previous Abortions[Title/Abstract])) OR (Abortion History[Title/Abstract])) OR (Abortion Histories[Title/Abstract])) OR (Histories, Abortion[Title/Abstract])) OR (History, Abortion[Title/Abstract])) OR (Abortion, Saline-Solution[Title/Abstract])) OR (Abortion, Saline Solution[Title/Abstract])) OR (Abortions, Saline-Solution[Title/Abstract])) OR (Saline-Solution Abortion[Title/Abstract])) OR (Saline-Solution Abortions[Title/Abstract])) OR (Abortion, Soap-Solution[Title/Abstract])) OR (Abortion, Soap Solution[Title/Abstract])) OR (Abortions, Soap-Solution[Title/Abstract])) OR (Soap-Solution Abortion[Title/Abstract])) OR (Soap-Solution Abortions[Title/Abstract])) OR (Anti-Abortion Groups[Title/Abstract])) OR (Anti Abortion Groups[Title/Abstract])) OR (Anti-Abortion Group[Title/Abstract])) OR (Group, Anti-Abortion[Title/Abstract])) OR (Groups, Anti-Abortion[Title/Abstract])) OR (Embryotomy[Title/Abstract])) OR (Embryotomies[Title/Abstract])) OR (Fertility Control, Postconception[Title/Abstract])) OR (Postconception Fertility Control[Title/Abstract])) OR (Abortion Failure[Title/Abstract])) OR (Abortion Failures[Title/Abstract])) OR (Failure, Abortion[Title/Abstract])) OR (Failures, Abortion[Title/Abstract])) OR (Abortion, Rivanol[Title/Abstract])) OR (Abortions, Rivanol[Title/Abstract])) OR (Rivanol Abortion[Title/Abstract])) OR (Rivanol Abortions[Title/Abstract])
#54	#52 OR #53
#55	"Hypertension, Pregnancy-Induced"[Mesh]
#56	((((((((((Hypertension, Pregnancy Induced[Title/Abstract]) OR (Pregnancy-Induced Hypertension[Title/Abstract])) OR (Pregnancy Induced Hypertension[Title/Abstract])) OR (Hypertensions, Pregnancy Induced[Title/Abstract])) OR (Induced Hypertension, Pregnancy[Title/Abstract]))

	OR (Induced Hypertensions, Pregnancy[Title/Abstract])) OR (Gestational Hypertension[Title/Abstract])) OR (Hypertension, Gestational[Title/Abstract])) OR (Transient Hypertension, Pregnancy[Title/Abstract])) OR (Hypertension, Pregnancy Transient[Title/Abstract])) OR (Pregnancy Transient Hypertension[Title/Abstract])
#57	#55 OR #56
#58	"Diabetes, Gestational"[Mesh]
#59	(((((Diabetes, Pregnancy-Induced[Title/Abstract]) OR (Diabetes, Pregnancy Induced[Title/Abstract])) OR (Pregnancy-Induced Diabetes[Title/Abstract])) OR (Gestational Diabetes[Title/Abstract])) OR (Diabetes Mellitus, Gestational[Title/Abstract])) OR (Gestational Diabetes Mellitus[Title/Abstract])
#60	#58 OR #59
#61	"Placenta Previa"[Mesh]
#62	Placenta Praevia[Title/Abstract]
#63	#61OR #62
#64	"Pre-Eclampsia"[Mesh]
#65	((((((((((((((((((((((((((((Pre Eclampsia[Title/Abstract]) OR (Pregnancy Toxemias[Title/Abstract])) OR (Pregnancy Toxemia[Title/Abstract])) OR (Toxemia, Pregnancy[Title/Abstract])) OR (Edema-Proteinuria-Hypertension Gestosis[Title/Abstract])) OR (Edema Proteinuria Hypertension Gestosis[Title/Abstract])) OR (Gestosis, Edema-Proteinuria-Hypertension[Title/Abstract])) OR (Hypertension-Edema-Proteinuria Gestosis[Title/Abstract])) OR (Gestosis, Hypertension-Edema-Proteinuria[Title/Abstract])) OR (Hypertension Edema Proteinuria Gestosis[Title/Abstract])) OR (Toxemia Of Pregnancy[Title/Abstract])) OR (Pregnancies, Toxemia Of[Title/Abstract])) OR (Pregnancy, Toxemia Of[Title/Abstract])) OR (Toxemia Of Pregnancies[Title/Abstract])) OR (EPH Complex[Title/Abstract])) OR (EPH Toxemias[Title/Abstract])) OR (EPH Toxemia[Title/Abstract])) OR (Toxemia, EPH[Title/Abstract])) OR (Toxemias, EPH[Title/Abstract])) OR (EPH Gestosis[Title/Abstract])) OR (Gestosis, EPH[Title/Abstract])) OR (Toxemias, Pregnancy[Title/Abstract])) OR (Preeclampsia[Title/Abstract])) OR (Proteinuria-Edema-Hypertension Gestosis[Title/Abstract])) OR (Gestosis, Proteinuria-Edema-Hypertension[Title/Abstract])) OR (Proteinuria Edema Hypertension Gestosis[Title/Abstract])) OR (Preeclampsia Eclampsia 1[Title/Abstract])) OR (1, Preeclampsia Eclampsia[Title/Abstract])) OR (1s, Preeclampsia Eclampsia[Title/Abstract])) OR (Eclampsia 1, Preeclampsia[Title/Abstract])) OR (Eclampsia 1s, Preeclampsia[Title/Abstract])) OR (Preeclampsia Eclampsia 1s[Title/Abstract])
#66	#64OR #65
#67	"Fetal Growth Retardation"[Mesh]
#68	(((Intrauterine Growth Retardation[Title/Abstract]) OR (Growth Retardation, Intrauterine[Title/Abstract])) OR (Intrauterine Growth Restriction[Title/Abstract])) OR (Fetal Growth Restriction[Title/Abstract])
#69	#67 OR #68
#70	"Premature Birth"[Mesh]
#71	((((((Birth, Premature[Title/Abstract]) OR (Births, Premature[Title/Abstract])) OR (Premature Births[Title/Abstract])) OR (Preterm Birth[Title/Abstract])) OR (Birth, Preterm[Title/Abstract])) OR (Births, Preterm[Title/Abstract])) OR (Preterm Births[Title/Abstract])

#72	#70 OR #71
#73	"Fetal Distress"[Mesh]
#74	(Nonreassuring Fetal Status[Title/Abstract]) OR (Fetal Status, Nonreassuring[Title/Abstract])
#75	#73 OR #74
#76	#48 OR #51 OR #54 OR #57 OR #60 OR #63 OR #66 OR #69 OR#72 OR #75
#77	#44 OR #45 OR #76
#78	meta-analysis[Publication Type] OR meta analysis[Title/Abstract] OR meta analysis[MeSH Terms] OR Systematic Review[Publication Type] OR Review, Systematic[Title/Abstract]
#79	#14 AND #77 AND #78

Table S2. Search strategy in the Embase (Ovid) database.

Search number	Query
#1	Antibodies, Antiphospholipid.mp. or exp phospholipid antibody/
#2	exp phospholipid antibody/ or Antiphospholipid Autoantibody.mp.
#3	exp phospholipid antibody/ or Autoantibody, Antiphospholipid.mp.
#4	Phospholipid Autoantibody.mp. or exp phospholipid antibody/
#5	exp phospholipid antibody/ or Autoantibody, Phospholipid.mp.
#6	Antiphospholipid Antibody.mp. or exp phospholipid antibody/
#7	exp phospholipid antibody/ or Antibody, Antiphospholipid.mp.
#8	Phospholipid Autoantibodies.mp. or exp phospholipid antibody/
#9	exp phospholipid antibody/ or Antiphospholipid Autoantibodies.mp.
#10	Antiphospholipid Antibodies.mp. or exp phospholipid antibody/
#11	1 or 2 or 3 or 4 or 5 or 6 or 7 or 8 or 9 or 10
#12	Antiphospholipid Syndrome.mp. or exp antiphospholipid syndrome/
#13	Syndrome, Antiphospholipid.mp. or exp antiphospholipid syndrome/
#14	Hughes Syndrome.mp. or exp antiphospholipid syndrome/
#15	exp antiphospholipid syndrome/ or Syndrome, Hughes.mp.
#16	Antiphospholipid Antibody Syndrome.mp. or exp antiphospholipid syndrome/
#17	exp antiphospholipid syndrome/ or Antibody Syndrome, Antiphospholipid.mp.
#18	Antiphospholipid Antibody Syndromes.mp. or exp antiphospholipid syndrome/
#19	exp antiphospholipid syndrome/ or Syndrome, Antiphospholipid Antibody.mp.
#20	Anti-Phospholipid Antibody Syndrome.mp. or exp antiphospholipid syndrome/
#21	Anti Phospholipid Antibody Syndrome.mp. or exp antiphospholipid syndrome/
#22	exp antiphospholipid syndrome/ or Antibody Syndrome, Anti-Phospholipid.mp.
#23	exp antiphospholipid syndrome/ or Syndrome, Anti-Phospholipid Antibody.mp.
#24	Anti-Phospholipid Syndrome.mp. or exp antiphospholipid syndrome/
#25	Anti Phospholipid Syndrome.mp. or exp antiphospholipid syndrome/
#26	exp antiphospholipid syndrome/ or Syndrome, Anti-Phospholipid.mp.
#27	12 or 13 or 14 or 15 or 16 or 17 or 18 or 19 or 20 or 21 or 22 or 23 or 24 or 25 or 26
#28	Lupus Erythematosus, Systemic.mp. or exp systemic lupus erythematosus/
#29	Systemic Lupus Erythematosus.mp. or exp systemic lupus erythematosus/
#30	Lupus Erythematosus Disseminatus.mp. or exp systemic lupus erythematosus/

#31	exp systemic lupus erythematosus/ or Libman-Sacks Disease.mp.
#32	exp systemic lupus erythematosus/ or Disease, Libman-Sacks.mp.
#33	exp systemic lupus erythematosus/ or Libman Sacks Disease.mp.
#34	exp systemic lupus erythematosus/ or systematic lupus erythematosus.mp.
#35	28 or 29 or 30 or 31 or 32 or 33 or 34
#36	SS-A antibodies.mp. or exp Ro antibody/
#37	exp Ro antibody/ or Sjogren's Anti-SS-A antibodies.mp.
#38	exp Ro antibody/ or Sjogren's Anti-SS-A autoantibody.mp.
#39	exp Ro antibody/ or Sjogren's Anti-SS-A autoantibodies.mp.
#40	SS A antibodies.mp. or exp Ro antibody/
#41	exp Ro antibody/ or Anti-Ro SSA autoantibodies.mp.
#42	anti-Ro52 autoantibodies.mp. or exp Sjoegren syndrome/
#43	Ro antibodies.mp. or exp Ro antibody/
#44	exp Ro antibody/ or Sjogren's Anti-SS-A antibody.mp.
#45	exp Ro antibody/ or Anti-Ro autoantibodies.mp.
#46	36 or 37 or 38 or 39 or 40 or 41 or 42 or 43 or 44 or 45
#47	exp La antibody/ or La antibod*.mp.
#48	exp antinuclear antibody/ or SSB autoantibod*.mp.
#49	SS B antibod*.mp. or exp Sjoegren syndrome/
#50	47 or 48 or 49
#51	11 or 27 or 35 or 46 or 50
#52	Ovarian Function Tests.mp. or exp ovarian function test/
#53	exp follitropin/ or basal follicle- stimulating hormone.mp.
#54	exp estrogen/ or basal estrogen.mp.
#55	antral follicle count.mp. or exp antral follicle count/
#56	52 or 53 or 54 or 55
#57	Ovarian Reserve.mp. or exp ovarian reserve/
#58	Ovarian Reserves.mp. or exp ovarian reserve/
#59	exp ovarian reserve/ or Reserve, Ovarian.mp.
#60	exp ovarian reserve/ or Reserves, Ovarian.mp.
#61	57 or 58 or 59 or 60
#62	Anti-Mullerian Hormone.mp. or exp Muellerian inhibiting factor/

#63	Anti Mullerian Hormone.mp. or exp Muellerian inhibiting factor/
#64	Mullerian-Inhibiting Factor.mp. or exp Muellerian inhibiting factor/
#65	Mullerian Inhibiting Factor.mp. or exp Muellerian inhibiting factor/
#66	exp Muellerian inhibiting factor/ or Anti-Mullerian Factor.mp.
#67	exp Muellerian inhibiting factor/ or Anti Mullerian Factor.mp.
#68	exp Muellerian inhibiting factor/ or Mullerian-Inhibitory Substance.mp.
#69	exp Muellerian inhibiting factor/ or Mullerian Inhibitory Substance.mp.
#70	exp Muellerian inhibiting factor/ or Mullerian Inhibiting Hormone.mp.
#71	Mullerian Inhibiting Substance.mp. or exp Muellerian inhibiting factor/
#72	exp Muellerian inhibiting factor/ or Mullerian Regression Factor.mp.
#73	exp Muellerian inhibiting factor/ or Mullerian-Inhibiting Hormone.mp.
#74	exp Muellerian inhibiting factor/ or Anti-Muellerian Hormone.mp.
#75	exp Muellerian inhibiting factor/ or Anti Muellerian Hormone.mp.
#76	exp Muellerian inhibiting factor/ or Hormone, Anti-Muellerian.mp.
#77	Antimullerian Hormone.mp. or exp Muellerian inhibiting factor/
#78	62 or 63 or 64 or 65 or 66 or 67 or 68 or 69 or 70 or 71 or 72 or 73 or 74 or 75 or 76 or 77
#79	Fertility.mp. or exp female fertility/
#80	exp female fertility/ or Fecundability.mp.
#81	Fecundity.mp. or exp fertility/
#82	exp female fertility/ or Fertility, Differential.mp.
#83	exp female infertility/ or Subfecundity.mp.
#84	exp female fertility/ or Fertility, Below Replacement.mp.
#85	exp fertility/ or Fertility, Marital.mp.
#86	Fertility, Natural.mp. or exp female fertility/
#87	79 or 80 or 81 or 82 or 83 or 84 or 85 or 86
#88	Infertility, Female.mp. or exp female infertility/
#89	exp fertility/ or Sterility, Postpartum.mp.
#90	exp fertility/ or Postpartum Sterility.mp.
#91	exp female subfertility/ or Subfertility, Female.mp.
#92	Female Subfertility.mp. or exp female subfertility/
#93	exp subfertility/ or Sub-Fertility, Female.mp.
#94	exp subfertility/ or Female Sub-Fertility.mp.

#95	Sterility, Female.mp. or exp female sterility/
#96	Female Sterility.mp. or exp female sterility/
#97	88 or 89 or 90 or 91 or 92 or 93 or 94 or 95 or 96
#98	1 or 2 or 3 or 4 or 5
#99	Reproductive Techniques, Assisted.mp. or exp infertility therapy/
#100	Assisted Reproductive Technique.mp. or exp infertility therapy/
#101	exp infertility therapy/ or Reproductive Technique, Assisted.mp.
#102	exp infertility therapy/ or Technique, Assisted Reproductive.mp.
#103	exp infertility therapy/ or Techniques, Assisted Reproductive.mp.
#104	exp infertility therapy/ or Assisted Reproductive Technics.mp.
#105	exp infertility therapy/ or Assisted Reproductive Technic.mp.
#106	exp embryo transfer/ or Reproductive Technic, Assisted.mp.
#107	exp infertility therapy/ or Reproductive Technics, Assisted.mp.
#108	exp embryo transfer/ or Technic, Assisted Reproductive.mp.
#109	exp infertility therapy/ or Technics, Assisted Reproductive.mp.
#110	Assisted Reproductive Techniques.mp. or exp infertility therapy/
#111	exp infertility therapy/ or Reproductive Technology, Assisted.mp.
#112	Assisted Reproductive Technologies.mp. or exp infertility therapy/
#113	Assisted Reproductive Technology.mp. or exp infertility therapy/
#114	exp infertility therapy/ or Reproductive Technologies, Assisted.mp.
#115	exp infertility therapy/ or Technologies, Assisted Reproductive.mp.
#116	exp infertility therapy/ or Technology, Assisted Reproductive.mp.
#117	99 or 100 or 101 or 102 or 103 or 104 or 105 or 106 or 107 or 108 or 109 or 110 or 111 or 112 or 113 or 114 or 115 or 116
#118	Sperm Injections, Intracytoplasmic.mp. or exp intracytoplasmic sperm injection/
#119	Injection, Intracytoplasmic Sperm.mp. or exp intracytoplasmic sperm injection/
#120	exp intracytoplasmic sperm injection/ or Injections, Intracytoplasmic Sperm.mp.
#121	Intracytoplasmic Sperm Injection.mp. or exp intracytoplasmic sperm injection/
#122	exp intracytoplasmic sperm injection/ or Sperm Injection, Intracytoplasmic.mp.
#123	Intracytoplasmic Sperm Injections.mp. or exp intracytoplasmic sperm injection/
#124	ICSI.mp. or exp intracytoplasmic sperm injection/
#125	exp intracytoplasmic sperm injection/ or Injections, Sperm, Intracytoplasmic.mp.
#126	118 or 119 or 120 or 121 or 122 or 123 or 124 or 125

#127	Fertilization in Vitro.mp. or exp in vitro fertilization/
#128	In Vitro Fertilization.mp. or exp in vitro fertilization/
#129	In Vitro Fertilizations.mp. or exp in vitro fertilization/
#130	exp in vitro fertilization/ or Test-Tube Fertilization.mp.
#131	exp in vitro fertilization/ or Fertilization, Test-Tube.mp.
#132	exp in vitro fertilization/ or Test Tube Fertilization.mp.
#133	exp in vitro fertilization/ or Test-Tube Fertilizations.mp.
#134	exp in vitro fertilization/ or Fertilizations in Vitro.mp.
#135	exp in vitro fertilization/ or Test-Tube Babies.mp.
#136	Babies, Test-Tube.mp. or exp in vitro fertilization/
#137	exp in vitro fertilization/ or Baby, Test-Tube.mp.
#138	exp in vitro fertilization/ or Test Tube Babies.mp.
#139	exp in vitro fertilization/ or Test-Tube Baby.mp.
#140	127 or 128 or 129 or 130 or 131 or 132 or 133 or 134 or 135 or 136 or 137 or 138 or 139
#141	117 or 126 or 140
#142	Pregnancy Outcome.mp. or exp pregnancy outcome/
#143	Pregnancy Outcomes.mp. or exp pregnancy outcome/
#144	exp pregnancy outcome/ or Outcome, Pregnancy.mp.
#145	exp pregnancy outcome/ or Outcomes, Pregnancy.mp.
#146	exp pregnancy rate/ or Clinical pregnancy rate.mp.
#147	exp live birth/ or live birth rate.mp.
#148	142 or 143 or 1444 or 145 or 146 or 147
#149	141 and 148
#150	Pregnancy Complications.mp. or exp pregnancy complication/
#151	Abortion, Spontaneous.mp. or exp spontaneous abortion/
#152	exp spontaneous abortion/ or Abortions, Spontaneous.mp.
#153	Spontaneous Abortions.mp. or exp spontaneous abortion/
#154	exp first trimester pregnancy/ or Early Pregnancy Loss.mp.
#155	exp first trimester pregnancy/ or Early Pregnancy Losses.mp.
#156	exp first trimester pregnancy/ or Loss, Early Pregnancy.mp.
#157	exp first trimester pregnancy/ or Losses, Early Pregnancy.mp.
#158	exp first trimester pregnancy/ or Pregnancy Loss, Early.mp.

#159	Pregnancy Losses, Early.mp. or exp first trimester pregnancy/
#160	Miscarriage.mp. or exp spontaneous abortion/
#161	Miscarriages.mp. or exp spontaneous abortion/
#162	Abortion, Tubal.mp. or exp ectopic pregnancy/
#163	Abortions, Tubal.mp. or exp ectopic pregnancy/
#164	Tubal Abortions.mp. or exp tubal abortion/
#165	151 or 152 or 153 or 154 or 155 or 156 or 157 or 158 or 159 or 160 or 161 or 162 or 163 or 164
#166	Abortion, Induced.mp. or exp induced abortion/
#167	exp induced abortion/ or Abortions, Induced.mp.
#168	Induced Abortions.mp. or exp induced abortion/
#169	Abortion, Drug-Induced.mp. or exp induced abortion/
#170	exp induced abortion/ or Abortion, Drug Induced.mp.
#171	exp induced abortion/ or Abortions, Drug-Induced.mp.
#172	exp induced abortion/ or Drug-Induced Abortions.mp.
#173	exp induced abortion/ or Abortion, Previous.mp.
#174	Abortions, Previous.mp. or exp induced abortion/
#175	Previous Abortions.mp. or exp previous abortion/
#176	Abortion, Saline-Solution.mp. or exp induced abortion/
#177	exp induced abortion/ or Abortion, Saline Solution.mp.
#178	exp induced abortion/ or Abortions, Saline-Solution.mp.
#179	exp induced abortion/ or Saline-Solution Abortions.mp.
#180	exp abortion/ or Abortion, Soap Solution.mp.
#181	exp induced abortion/ or Group, Anti-Abortion.mp.
#182	Groups, Anti-Abortion.mp. or exp induced abortion/
#183	exp induced abortion/ or Embryotomy.mp.
#184	exp induced abortion/ or Embryotomies.mp.
#185	exp induced abortion/ or Fertility Control, Postconception.mp.
#186	exp induced abortion/ or Abortion, Rivanol.mp.
#187	exp induced abortion/ or Abortions, Rivanol.mp.
#188	Rivanol Abortions.mp. or exp induced abortion/
#189	166 or 167 or 168 or 169 or 170 or 171 or 172 or 173 or 174 or 175 or 176 or 177 or 178 or 179 or 180 or 181 or 182 or 183 or 184 or 185 or 186 or 187 or 188
#190	Hypertension, Pregnancy-Induced.mp. or exp maternal hypertension/

#191	Hypertension, Pregnancy Induced.mp. or exp maternal hypertension/
#192	Pregnancy-Induced Hypertension.mp. or exp maternal hypertension/
#193	Pregnancy Induced Hypertension.mp. or exp maternal hypertension/
#194	exp maternal hypertension/ or Hypertensions, Pregnancy Induced.mp.
#195	exp maternal hypertension/ or Induced Hypertension, Pregnancy.mp.
#196	exp maternal hypertension/ or Induced Hypertensions, Pregnancy.mp.
#197	Gestational Hypertension.mp. or exp maternal hypertension/
#198	exp maternal hypertension/ or Hypertension, Gestational.mp.
#199	exp preeclampsia/ or Transient Hypertension, Pregnancy.mp.
#200	exp preeclampsia/ or Hypertension, Pregnancy Transient.mp.
#201	exp maternal hypertension/ or Pregnancy Transient Hypertension.mp.
#202	190 or 191 or 192 or 193 or 194 or 195 or 196 or 198 or 198 or 199 or 200 or 201
#203	Diabetes, Gestational.mp. or exp gestational diabetes/
#204	exp pregnancy diabetes mellitus/ or Diabetes, Pregnancy-Induced.mp.
#205	exp pregnancy diabetes mellitus/ or Diabetes, Pregnancy Induced.mp.
#206	Pregnancy-Induced Diabetes.mp. or exp gestational diabetes/
#207	Gestational Diabetes.mp. or exp pregnancy diabetes mellitus/
#208	exp pregnancy diabetes mellitus/ or Diabetes Mellitus, Gestational.mp.
#209	Gestational Diabetes Mellitus.mp. or exp gestational diabetes/
#210	203 or 204 or 205 or 206 or 207 or 208 or 209
#211	Placenta Previa.mp. or exp placenta previa/
#212	Placenta Praevia.mp. or exp placenta previa/
#213	211 or 212
#214	Pre-Eclampsia.mp. or exp preeclampsia/
#215	Pre Eclampsia.mp. or preeclampsia/
#216	Pregnancy Toxemias.mp. or exp preeclampsia/
#217	Pregnancy Toxemia.mp. or exp preeclampsia/
#218	exp preeclampsia/ or Toxemia, Pregnancy.mp.
#219	Edema-Proteinuria-Hypertension Gestosis.mp. or exp preeclampsia/
#220	Edema Proteinuria Hypertension Gestosis.mp. or exp preeclampsia/
#221	exp preeclampsia/ or Gestosis, Edema-Proteinuria-Hypertension.mp.
#222	exp maternal hypertension/ or Hypertension-Edema-Proteinuria Gestosis.mp.

#223	exp maternal hypertension/ or Gestosis, Hypertension-Edema-Proteinuria.mp.
#224	exp preeclampsia/ or Hypertension Edema Proteinuria Gestosis.mp.
#225	exp preeclampsia/ or Toxemia Of Pregnancy.mp.
#226	exp preeclampsia/ or Of Pregnancy, Toxemia.mp.
#227	exp preeclampsia/ or Pregnancies, Toxemia Of.mp.
#228	exp preeclampsia/ or Pregnancy, Toxemia Of.mp.
#229	exp preeclampsia/ or Toxemia Of Pregnancies.mp.
#230	EPH Toxemias.mp. or exp preeclampsia/
#231	EPH Toxemia.mp. or exp preeclampsia/
#232	exp preeclampsia/ or Toxemia, EPH.mp.
#233	EPH Gestosis.mp. or exp preeclampsia/
#234	Gestosis, EPH.mp. or exp preeclampsia/
#235	exp preeclampsia/ or Toxemias, Pregnancy.mp.
#236	exp preeclampsia/ or Preeclampsia.mp.
#237	exp preeclampsia/ or Proteinuria Edema Hypertension Gestosis.mp.
#238	214 or 215 or 216 or 217 or 218 or 219 or 220 or 221 or 222 or 223 or 224 or 225 or 226 or 227 or 228 or 229 or 230 or 231 or 232 or 233 or 234 or 235 or 236 or 237
#239	Fetal Growth Retardation.mp. or exp intrauterine growth retardation/
#240	Intrauterine Growth Retardation.mp. or exp intrauterine growth retardation/
#241	intrauterine growth retardation/ or Growth Retardation, Intrauterine.mp.
#242	Intrauterine Growth Restriction.mp. or exp intrauterine growth retardation/
#243	Fetal Growth Restriction.mp. or exp intrauterine growth retardation/
#244	239 or 240 or 241 or 242 or 243
#245	Premature Birth.mp. or exp prematurity/
#246	Birth, Premature.mp. or exp prematurity/
#247	Births, Premature.mp. or exp premature labor/
#248	Premature Births.mp. or exp prematurity/
#249	Preterm Birth.mp. or exp prematurity/
#250	exp premature labor/ or Birth, Preterm.mp.
#251	Births, Preterm.mp. or exp premature labor/
#252	Preterm Births.mp. or exp prematurity/
#253	245 or 246 or 247 or 248 or 249 or 250 or 251 or 252
#254	Fetal Distress.mp. or exp fetus distress/

#255	Nonreassuring Fetal Status.mp. or exp fetus distress/
#256	exp fetus distress/ or Fetal Status, Nonreassuring.mp.
#257	254 or 255 or 256
#258	150 or 165 or 189 or 202 or 210 or 213 or 238 or 244 or 253 or 257
#259	98 or 149 or 258
#260	(meta-analysis or systematic review).tw.
#261	51 and 259 and 260
#262	limit female
#263	limit English
#264	remove preprint records
#265	limit human

Table S3. Search strategy in the Cochrane database of systematic reviews.

Search number	Query
#1	MeSH descriptor: [Antibodies, Antiphospholipid] explode all trees
#2	(Antiphospholipid Autoantibody):ti,ab,kw OR (Autoantibody, Antiphospholipid):ti,ab,kw OR (Phospholipid Autoantibody):ti,ab,kw OR (Autoantibody, Phospholipid):ti,ab,kw OR (Antiphospholipid Antibody):ti,ab,kw
#3	(Antibody, Antiphospholipid):ti,ab,kw OR (Phospholipid Autoantibodies):ti,ab,kw OR (Antiphospholipid Autoantibodies):ti,ab,kw OR (Antiphospholipid Antibodies):ti,ab,kw
#4	#1 OR #2 OR #3
#5	MeSH descriptor: [Antiphospholipid Syndrome] explode all trees
#6	(Syndrome, Antiphospholipid):ti,ab,kw OR (Hughes Syndrome):ti,ab,kw OR (Syndrome, Hughes):ti,ab,kw OR (Antiphospholipid Antibody Syndrome):ti,ab,kw OR (Antibody Syndrome, Antiphospholipid):ti,ab,kw
#7	(Antiphospholipid Antibody Syndromes):ti,ab,kw OR (Syndrome, Antiphospholipid Antibody):ti,ab,kw OR (Anti-Phospholipid Antibody Syndrome):ti,ab,kw OR (Anti Phospholipid Antibody Syndrome):ti,ab,kw OR (Antibody Syndrome, Anti-Phospholipid):ti,ab,kw
#8	(Syndrome, Anti-Phospholipid Antibody):ti,ab,kw OR (Anti-Phospholipid Syndrome):ti,ab,kw OR (Anti Phospholipid Syndrome):ti,ab,kw OR (Syndrome, Anti-Phospholipid):ti,ab,kw
#9	#5 OR #6 OR #7 OR #8
#10	MeSH descriptor: [Lupus Erythematosus, Systemic] explode all trees
#11	(Systemic Lupus Erythematosus):ti,ab,kw OR (Lupus Erythematosus Disseminatus):ti,ab,kw OR (Libman-Sacks Disease):ti,ab,kw OR (Disease, Libman-Sacks):ti,ab,kw OR (Libman Sacks Disease):ti,ab,kw
#12	(systematic lupus erythematosus):ti,ab,kw
#13	#10 OR #11 OR #12
#14	Any MeSH descriptor in all MeSH products
#15	(Sjogren's Anti-SS-A antibodies):ti,ab,kw OR (Sjogren's Anti-SS-A autoantibody):ti,ab,kw OR (Sjogren's Anti-SS-A autoantibodies):ti,ab,kw OR (SS A antibodies):ti,ab,kw OR (Anti-Ro SSA autoantibodies):ti,ab,kw
#16	(Anti-SSA autoantibodies):ti,ab,kw OR (anti-Ro52 autoantibodies):ti,ab,kw OR (anti-SSA Ro52 autoantibodies):ti,ab,kw OR (Ro antibodies):ti,ab,kw OR (Sjogren's Anti-SS-A antibody):ti,ab,kw
#17	(Anti-Ro autoantibodies):ti,ab,kw
#18	#14 OR #15 OR #16 OR #17
#19	(SS B antibod*):ti,ab,kw OR (SSB autoantibod*):ti,ab,kw OR (La antibod*):ti,ab,kw OR (La autoantibod*):ti,ab,kw
#20	#4 OR #9 OR #13 OR #18 OR #19
#21	MeSH descriptor: [Ovarian Function Tests] explode all trees
#22	(Function Test, Ovarian):ti,ab,kw OR (Function Tests, Ovarian):ti,ab,kw OR (Ovarian Function Test):ti,ab,kw OR (Test, Ovarian

	Function):ti,ab,kw OR (Tests, Ovarian Function):ti,ab,kw
#23	(basal follicle stimulating hormone):ti,ab,kw OR (basal estrogen):ti,ab,kw OR (antral follicle count):ti,ab,kw
#24	#21 OR #22 OR #23
#25	MeSH descriptor: [Ovarian Reserve] explode all trees
#26	(Ovarian Reserves):ti,ab,kw OR (Reserve, Ovarian):ti,ab,kw OR (Reserves, Ovarian):ti,ab,kw
#27	#25 OR #26
#28	MeSH descriptor: [Anti-Mullerian Hormone] explode all trees
#29	(Anti Mullerian Hormone):ti,ab,kw OR (Mullerian-Inhibiting Factor):ti,ab,kw OR (Mullerian Inhibiting Factor):ti,ab,kw OR (Anti-Mullerian Factor):ti,ab,kw OR (Anti Mullerian Factor):ti,ab,kw
#30	(Mullerian-Inhibitory Substance):ti,ab,kw OR (Mullerian Inhibitory Substance):ti,ab,kw OR (Mullerian Inhibiting Hormone):ti,ab,kw OR (Mullerian Inhibiting Substance):ti,ab,kw OR (Mullerian Regression Factor):ti,ab,kw
#31	(Mullerian-Inhibiting Hormone):ti,ab,kw OR (Anti-Muellerian Hormone):ti,ab,kw OR (Anti Muellerian Hormone):ti,ab,kw OR (Hormone, Anti-Muellerian):ti,ab,kw OR (Antimullerian Hormone):ti,ab,kw
#32	#28 OR #29 OR #30 OR #31
#33	MeSH descriptor: [Fertilizers] in all MeSH products
#34	(Fecundability):ti,ab,kw OR (Fecundity):ti,ab,kw OR (Differential Fertility):ti,ab,kw OR (Fertility, Differential):ti,ab,kw OR (Fertility Determinants):ti,ab,kw
#35	(Determinant, Fertility):ti,ab,kw OR (Determinants, Fertility):ti,ab,kw OR (Fertility Determinant):ti,ab,kw OR (Subfecundity):ti,ab,kw OR (Fertility Preferences):ti,ab,kw
#36	(Fertility Preference):ti,ab,kw OR (Preference, Fertility):ti,ab,kw OR (Preferences, Fertility):ti,ab,kw OR (Fertility, Below Replacement):ti,ab,kw OR (Below Replacement Fertility):ti,ab,kw
#37	(Marital Fertility):ti,ab,kw OR (Fertility, Marital):ti,ab,kw OR (Natural Fertility):ti,ab,kw OR (Fertility, Natural):ti,ab,kw OR (World Fertility Survey):ti,ab,kw
#38	(Fertility Survey, World):ti,ab,kw OR (Fertility Surveys, World):ti,ab,kw OR (Survey, World Fertility):ti,ab,kw OR (Surveys, World Fertility):ti,ab,kw OR (World Fertility Surveys):ti,ab,kw
#39	(Fertility Incentives):ti,ab,kw OR (Fertility Incentive):ti,ab,kw
#40	#33 OR #34 OR #35 OR #36 OR #37 OR #38 OR #39
#41	MeSH descriptor: [Infertility, Female] explode all trees
#42	(Female Infertility):ti,ab,kw OR (Sterility, Postpartum):ti,ab,kw OR (Postpartum Sterility):ti,ab,kw OR (Subfertility, Female):ti,ab,kw OR (Female Subfertility):ti,ab,kw
#43	(Sub-Fertility, Female):ti,ab,kw OR (Female Sub-Fertility):ti,ab,kw OR (Sub Fertility, Female):ti,ab,kw OR (Sterility, Female):ti,ab,kw OR (Female Sterility):ti,ab,kw
#44	#41 OR #42 OR #43

#45	MeSH descriptor: [Reproductive Techniques, Assisted] explode all trees
#46	(Assisted Reproductive Technique):ti,ab,kw OR (Reproductive Technique, Assisted):ti,ab,kw OR (Technique, Assisted Reproductive):ti,ab,kw OR (Techniques, Assisted Reproductive):ti,ab,kw OR (Assisted Reproductive Technics):ti,ab,kw
#47	(Assisted Reproductive Technic):ti,ab,kw OR (Reproductive Technic, Assisted):ti,ab,kw OR (Reproductive Technics, Assisted):ti,ab,kw OR (Technic, Assisted Reproductive):ti,ab,kw OR (Technics, Assisted Reproductive):ti,ab,kw
#48	(Assisted Reproductive Techniques):ti,ab,kw OR (Reproductive Technology, Assisted):ti,ab,kw OR (Assisted Reproductive Technologies):ti,ab,kw OR (Assisted Reproductive Technology):ti,ab,kw OR (Reproductive Technologies, Assisted):ti,ab,kw
#49	(Technologies, Assisted Reproductive):ti,ab,kw OR (Technology, Assisted Reproductive):ti,ab,kw
#50	45 OR #46 OR #47 OR #48 OR #49
#51	MeSH descriptor: [Sperm Injections, Intracytoplasmic] explode all trees
#52	(Injection, Intracytoplasmic Sperm):ti,ab,kw OR (Injections, Intracytoplasmic Sperm):ti,ab,kw OR (Intracytoplasmic Sperm Injection):ti,ab,kw OR (Sperm Injection, Intracytoplasmic):ti,ab,kw OR (Intracytoplasmic Sperm Injections):ti,ab,kw
#53	(ICSI):ti,ab,kw OR (Injections, Sperm, Intracytoplasmic):ti,ab,kw
#54	#51 OR #52 OR #53
#55	MeSH descriptor: [Fertilization in Vitro] explode all trees
#56	(In Vitro Fertilization):ti,ab,kw OR (In Vitro Fertilizations):ti,ab,kw OR (Test-Tube Fertilization):ti,ab,kw OR (Fertilization, Test-Tube):ti,ab,kw OR (Fertilizations, Test-Tube):ti,ab,kw
#57	(Test Tube Fertilization):ti,ab,kw OR (Test-Tube Fertilizations):ti,ab,kw OR (Fertilizations in Vitro):ti,ab,kw OR (Test-Tube Babies):ti,ab,kw OR (Babies, Test-Tube):ti,ab,kw
#58	(Baby, Test-Tube):ti,ab,kw OR (Test Tube Babies):ti,ab,kw OR (Test-Tube Baby):ti,ab,kw
#59	#55 OR #56 OR #57 OR #58
#60	MeSH descriptor: [Pregnancy Outcome] explode all trees
#61	(Pregnancy Outcomes):ti,ab,kw OR (Outcome, Pregnancy):ti,ab,kw OR (Outcomes, Pregnancy):ti,ab,kw
#62	(Clinical pregnancy rate):ti,ab,kw OR (live birth rate):ti,ab,kw
#63	#60 OR #61 OR #62
#64	#50 OR #54 OR #59
#65	#63 AND #64
#66	#24 OR #27 OR #32 OR #40 OR #44
#67	MeSH descriptor: [Pregnancy Complications] explode all trees
#68	(Complication, Pregnancy):ti,ab,kw OR (Pregnancy Complication):ti,ab,kw OR (Complications, Pregnancy):ti,ab,kw OR (Adverse Birth Outcomes):ti,ab,kw OR (Adverse Birth Outcome):ti,ab,kw
#69	(Birth Outcome, Adverse):ti,ab,kw OR (Outcome, Adverse Birth):ti,ab,kw
#70	#67 OR #68 OR #69

#71	MeSH descriptor: [Abortion, Spontaneous] explode all trees
#72	(Abortions, Spontaneous):ti,ab,kw OR (Spontaneous Abortions):ti,ab,kw OR (Spontaneous Abortion):ti,ab,kw OR (Early Pregnancy Loss):ti,ab,kw OR (Early Pregnancy Losses):ti,ab,kw
#73	(Loss, Early Pregnancy):ti,ab,kw OR (Losses, Early Pregnancy):ti,ab,kw OR (Pregnancy Loss, Early):ti,ab,kw OR (Pregnancy Losses, Early):ti,ab,kw OR (Miscarriage):ti,ab,kw
#74	(Miscarriages):ti,ab,kw OR (Abortion, Tubal):ti,ab,kw OR (Abortions, Tubal):ti,ab,kw OR (Tubal Abortion):ti,ab,kw OR (Tubal Abortions):ti,ab,kw
#75	#71 OR #72 OR #73 OR #74
#76	MeSH descriptor: [Abortion, Induced] explode all trees
#77	(Induced Abortion):ti,ab,kw OR (Abortions, Induced):ti,ab,kw OR (Induced Abortions):ti,ab,kw OR (Abortion (Induced)):ti,ab,kw OR (Abortions (Induced)):ti,ab,kw
#78	(Abortion Rate):ti,ab,kw OR (Abortion Rates):ti,ab,kw OR (Rate, Abortion):ti,ab,kw OR (Rates, Abortion):ti,ab,kw OR (Abortion Techniques):ti,ab,kw
#79	(Abortion Technique):ti,ab,kw OR (Technique, Abortion):ti,ab,kw OR (Techniques, Abortion):ti,ab,kw OR (Abortion Technics):ti,ab,kw OR (Abortion Technic):ti,ab,kw
#80	(Technic, Abortion):ti,ab,kw OR (Technics, Abortion):ti,ab,kw OR (Abortion, Drug-Induced):ti,ab,kw OR (Abortion, Drug Induced):ti,ab,kw OR (Abortions, Drug-Induced):ti,ab,kw
#81	(Drug-Induced Abortion):ti,ab,kw OR (Drug-Induced Abortions):ti,ab,kw OR (Previous Abortion):ti,ab,kw OR (Abortion, Previous):ti,ab,kw OR (Abortions, Previous):ti,ab,kw
#82	(Previous Abortions):ti,ab,kw OR (Abortion History):ti,ab,kw OR (Abortion Histories):ti,ab,kw OR (Histories, Abortion):ti,ab,kw OR (History, Abortion):ti,ab,kw
#83	(Abortion, Saline-Solution):ti,ab,kw OR (Abortion, Saline Solution):ti,ab,kw OR (Abortions, Saline-Solution):ti,ab,kw OR (Saline-Solution Abortion):ti,ab,kw OR (Saline-Solution Abortions):ti,ab,kw
#84	(Abortion, Soap-Solution):ti,ab,kw OR (Abortion, Soap Solution):ti,ab,kw OR (Abortions, Soap-Solution):ti,ab,kw OR (Soap-Solution Abortion):ti,ab,kw OR (Soap-Solution Abortions):ti,ab,kw
#85	(Anti-Abortion Groups):ti,ab,kw OR (Anti Abortion Groups):ti,ab,kw OR (Anti-Abortion Group):ti,ab,kw OR (Group, Anti-Abortion):ti,ab,kw OR (Groups, Anti-Abortion):ti,ab,kw
#86	(Abortion Failures):ti,ab,kw OR (Failure, Abortion):ti,ab,kw OR (Failures, Abortion):ti,ab,kw OR (Abortion, Rivanol):ti,ab,kw OR (Abortions, Rivanol):ti,ab,kw
#87	(Rivanol Abortion):ti,ab,kw OR (Rivanol Abortions):ti,ab,kw
#88	(Embryotomy):ti,ab,kw OR (Embryotomies):ti,ab,kw OR (Fertility Control, Postconception):ti,ab,kw OR (Postconception Fertility Control):ti,ab,kw OR (Abortion Failure):ti,ab,kw
#89	#76 OR #77 OR #78 OR #79 OR #80 OR #81

#90	#82 OR #83 OR #85
#91	#86 OR #87 OR #88
#92	#89 OR #90 OR #91
#93	MeSH descriptor: [Hypertension, Pregnancy-Induced] explode all trees
#94	(Hypertension, Pregnancy Induced):ti,ab,kw OR (Pregnancy-Induced Hypertension):ti,ab,kw OR (Pregnancy Induced Hypertension):ti,ab,kw OR (Hypertensions, Pregnancy Induced):ti,ab,kw OR (Induced Hypertension, Pregnancy):ti,ab,kw
#95	(Induced Hypertensions, Pregnancy):ti,ab,kw OR (Gestational Hypertension):ti,ab,kw OR (Hypertension, Gestational):ti,ab,kw OR (Transient Hypertension, Pregnancy):ti,ab,kw OR (Hypertension, Pregnancy Transient):ti,ab,kw
#96	(Pregnancy Transient Hypertension):ti,ab,kw
#97	#93 OR #94 OR #95 OR #96
#98	MeSH descriptor: [Diabetes, Gestational] explode all trees
#99	(Diabetes, Pregnancy-Induced):ti,ab,kw OR (Diabetes, Pregnancy Induced):ti,ab,kw OR (Pregnancy-Induced Diabetes):ti,ab,kw
#100	(Gestational Diabetes):ti,ab,kw OR (Diabetes Mellitus, Gestational):ti,ab,kw OR (Gestational Diabetes Mellitus):ti,ab,kw
#101	#98 OR #99 OR #100
#102	MeSH descriptor: [Placenta Previa] explode all trees
#103	(Placenta Praevia):ti,ab,kw
#104	#102 OR #103
#105	MeSH descriptor: [Pre-Eclampsia] explode all trees
#106	(Pre Eclampsia):ti,ab,kw OR (Pregnancy Toxemias):ti,ab,kw OR (Pregnancy Toxemia):ti,ab,kw OR (Toxemia, Pregnancy):ti,ab,kw OR (Edema-Proteinuria-Hypertension Gestosis):ti,ab,kw
#107	(Edema Proteinuria Hypertension Gestosis):ti,ab,kw OR (Gestosis, Edema-Proteinuria-Hypertension):ti,ab,kw OR (Hypertension-Edema-Proteinuria Gestosis):ti,ab,kw OR (Gestosis, Hypertension-Edema-Proteinuria):ti,ab,kw OR (Hypertension Edema Proteinuria Gestosis):ti,ab,kw
#108	(Toxemia Of Pregnancy):ti,ab,kw OR (Of Pregnancies, Toxemia):ti,ab,kw OR (Of Pregnancy, Toxemia):ti,ab,kw OR (Pregnancies, Toxemia Of):ti,ab,kw OR (Pregnancy, Toxemia Of):ti,ab,kw
#109	(Toxemia Of Pregnancies):ti,ab,kw OR (EPH Complex):ti,ab,kw OR (EPH Toxemias):ti,ab,kw OR (EPH Toxemia):ti,ab,kw OR (Toxemia, EPH):ti,ab,kw
#110	(Toxemias, EPH):ti,ab,kw OR (EPH Gestosis):ti,ab,kw OR (Gestosis, EPH):ti,ab,kw OR (Toxemias, Pregnancy):ti,ab,kw OR (Preeclampsia):ti,ab,kw
#111	(Proteinuria-Edema-Hypertension Gestosis):ti,ab,kw OR (Gestosis, Proteinuria-Edema-Hypertension):ti,ab,kw OR (Proteinuria Edema Hypertension Gestosis):ti,ab,kw OR (Preeclampsia Eclampsia 1):ti,ab,kw OR (1, Preeclampsia Eclampsia):ti,ab,kw
#112	(1s, Preeclampsia Eclampsia):ti,ab,kw OR (Eclampsia 1, Preeclampsia):ti,ab,kw OR (Eclampsia 1s, Preeclampsia):ti,ab,kw OR (Preeclampsia Eclampsia 1s):ti,ab,kw

#113	#105 OR #106 OR #107 OR #108 OR #109 OR #110 OR #111 OR #112
#114	MeSH descriptor: [Fetal Growth Retardation] explode all trees
#115	(Intrauterine Growth Retardation):ti,ab,kw OR (Growth Retardation, Intrauterine):ti,ab,kw OR (Intrauterine Growth Restriction):ti,ab,kw OR (Fetal Growth Restriction):ti,ab,kw
#116	#114 OR #115
#117	MeSH descriptor: [Premature Birth] explode all trees
#118	(Birth, Premature):ti,ab,kw OR (Births, Premature):ti,ab,kw OR (Premature Births):ti,ab,kw OR (Preterm Birth):ti,ab,kw
#119	(Birth, Preterm):ti,ab,kw OR (Births, Preterm):ti,ab,kw OR (Preterm Births):ti,ab,kw
#120	#117 OR #118 OR #119
#121	MeSH descriptor: [Fetal Distress] explode all trees
#122	(Nonreassuring Fetal Status):ti,ab,kw OR (Fetal Status, Nonreassuring):ti,ab,kw
#123	#121 OR #122
#124	#70 OR #75 OR #92
#125	#97 OR #101 OR #104
#126	#113 OR #116
#127	#120 OR #123
#128	#124 OR #125
#129	#126 OR #127
#130	#128 OR #129
#131	#65 OR #66 OR #130
#132	#20 AND #131

Table S4. Quality assessment of included articles using AMSTAR 2 tool.

	1	2	3	4	5	6	7	8	9	10	11	12	13	14	15	16	Total quality
Upala, 2016	Yes	Yes	Yes	Partial yes	Yes	Yes	Partial yes	Yes	Yes	Yes	Yes	Yes	Yes	Yes	Yes	Yes	Moderate
Luo, 2020	Yes	Partial yes	No	Partial yes	Yes	No	No	Partial yes	Yes	No	Yes	Yes	Yes	Yes	Yes	Yes	Low
Bundhun, 2017	Yes	Partial yes	Yes	Yes	No	No	Yes	Partial yes	Yes	Yes	Yes	Yes	Yes	Yes	Yes	Yes	Low
Prado, 2010	Yes	Yes	Yes	Yes	Yes	Yes	Yes	Yes	Yes	No	Yes	Yes	Yes	Yes	Yes	Yes	Moderate
Wei, 2017	Yes	Partial yes	Yes	Partial yes	Yes	Yes	No	Partial yes	Yes	Yes	Yes	Yes	Yes	Yes	Yes	Yes	Moderate
Tan, 2022	Yes	partial yes	Yes	Partial yes	No	No	No	Yes	Yes	Yes	Yes	Yes	Yes	Yes	Yes	Yes	Low
Gao, 2021	Yes	Partial yes	Yes	Partial yes	Yes	Yes	Partial yes	Yes	Yes	Yes	Yes	Yes	Yes	Yes	Yes	Yes	Moderate
Liu, 2019	Yes	Partial yes	Yes	Partial yes	Yes	Yes	Partial yes	Yes	Partial yes	No	Yes	Yes	Yes	Yes	Yes	Yes	Moderate
Nassar, 2011	Yes	Partial yes	Yes	Partial yes	Yes	Yes	Partial yes	Partial yes	No	No	Yes	No	Yes	Yes	No	No	Low
Xu, 2022	Yes	Yes	Yes	Partial yes	Yes	Yes	Partial Yes	Partial yes	Partial yes	No	Yes	Yes	Yes	Yes	Yes	Yes	Moderate
Dong (Dai), 2019	Yes	Partial yes	Yes	Partial yes	Yes	Yes	Partial yes	Partial yes	No	Yes	Yes	Yes	Yes	Yes	No	Yes	Low
Wen He, 2020	Yes	Partial yes	No	Partial yes	Yes	Yes	Partial yes	Yes	Partial yes	No	Yes	Yes	Yes	Yes	Yes	Yes	Low
Dong (Yuan), 2019	Yes	Partial yes	Yes	Partial yes	Yes	Yes	Partial yes	Yes	Partial yes	Yes	Yes	Yes	Yes	Yes	Yes	Yes	Moderate
Geng, 2022	Yes	Yes	Yes	Yes	Yes	Yes	Yes	Yes	Yes	Yes	Yes	Yes	Yes	Yes	Yes	Yes	Moderate
Hornstein, 2000	Yes	Partial yes	Yes	Partial yes	No	No	Yes	Partial yes	No	No	Yes	No	No	Yes	No	No	Very low
Simopoulou, 2019	Yes	No	Yes	Partial yes	Yes	Yes	No	Partial yes	No	No	Yes	No	No	No	No	Yes	Very low

Table S5. Overlapping between articles for the association between SLE and spontaneous abortion.

	Bundhun, 2017	He, 2020
Barnado, 2014	√	
Mcgrory, 2003	√	
Molokhia, 2007	√	
Yuen, 2008	√	
Ling, 2018		√
Wu, 2018		√
Abdwani, 2018		√
CCA=0%		

Table S6. Overlapping between articles for the association between SLE and PE.

	Bundhun, 2017	He Wen, 2020	Dong (Yuan), 2019
Arkema, 2015	√		
Barnabe, 2011	√		√
Barnado, 2014	√		√
Jackobsen, 2015	√		√
Mcgrory, 2003	√		
Wallenius, 2014	√		√
Abdwani, 2018		√	√
Bandoli, 2019		√	
Ling, 2018		√	√
Phansenee, 2017		√	√
Wu, 2018		√	√
Hamed, 2013			√
Simard, 2017			√
CCA=30.76%			

Table S7. Overlapping between articles for the association between SLE and GDM.

	Dong (Dai), 2019	He Wen, 2020
Abdwani, 2018	√	√
Galappatthy, 2017	√	
Phansenee, 2017	√	√
Wu, 2018	√	√
Yuen, 2008	√	
Bandoli, 2019		√
CCA=50%		

Table S8. Overlapping between articles for the association between SLE and premature delivery.

	Bundhun, 2017	Wei, 2017	He, 2020
Arkema, 2015	√		
Barnabe, 2011	√		
Barnado, 2014	√	√	
Jackobsen, 2015	√		
Molokhia, 2007		√	
Wallenius, 2014	√		
Yuen, 2008	√	√	
Georgiou, 2000		√	
Hamed, 2013		√	
Julkunen, 1993		√	
Abdwani, 2018			√
Bandoli, 2019			√
Gnacio, 2018			√
Ling, 2018			√
Phansenee, 2017			√
Wu, 2018			√
CCA=6.25%			

Table S9. Overlapping between articles for the association between SLE and SGA.

	Bundhun, 2017	He, 2020
Barnabe, 2011	√	
Chen, 2010	√	
Nili, 2013	√	
Wallenius, 2014	√	
Bandoli, 2019		√
Gnacio, 2018		√
Wu, 2018		√
Phensenee, 2017		√
CCA=0%		

Table S10. Overlapping between articles for the association between SLE and LBW.

	Bundhun, 2017	He, 2020
Arkema, 2015	√	
Wallenius, 2014	√	
Abdwani, 2018		√
Gnacio, 2018		√
Phansenee, 2017		√
CCA=0%		

Table S11. Overlapping between articles for the association between SLE and NICU.

	Bundhun, 2017	He, 2020
Barnabe, 2011	√	
Nili, 2013	√	
Wallenius, 2014	√	
Gnacio, 2018		√
Wu, 2018		√
CCA=0%		

Table S12. Overlapping between articles for the association between SLE and One minute Apgar<7.

	Bundhun, 2017	He, 2020
Arkema, 2015	√	
Nili, 2013	√	
Wallenius, 2014	√	
Abdwani, 2018		√
Phansenee, 2017		√
Wu, 2018		√
CCA=0%		

Table S13. Overlapping between articles for the association between pSS and premature delivery.

	Upala, 2016	Geng, 2022
Carolis, 2014	√	√
Takaya, 2011	√	
Mavridou, 2010	√	
Skopouli, 2008	√	
Hussein, 2011	√	√
Ballester, 2017		√
L Li, 2019		√
Priori, 2013		√
Elliott, 2019		√
Yang, 2021		√
Haga, 2005		√
Xu, 2019		√
CCA=16.67%		

Table S14. Overlapping between articles for the association between aPLs and PE.

	Doprado, 2010	Nassar, 2011
Allen, 1996	√	√
Bowen, 2002	√	√
Branch, 1989	√	
Dreyfus, 2001	√	√
Facchinetti, 2009	√	
Faden, 1997	√	√
Harris, 1991	√	
Kupfermine, 2000	√	
Lee, 2003	√	√
Mello, 2005	√	
Pattison, 1993	√	√
Yasuda, 1995	√	√
Katano, 1996		√
Lynch, 1995		√
Matthiesen, 1999		√
Mello, 1999		√
Moodley, 1995		√
Rix, 1992		√
Schjetlein, 1998		√
Stuart, 1993		√
Uncu, 1996		√
Vahid, 2006		√
Macho, 2002		√
Vanpampus, 1999		√
Yamamoto, 1996		√
Alfirevic, 2001		√
CCA=26.92%		

Table S15. Overlapping between articles for the association between aPLs and IUGR.

	Xu, 2022	Nassar, 2011
Alfirevic, 2001	√	√
Reig, 2019	√	
Berks, 2015	√	
Bouvier, 2013	√	
Canti, 2011	√	
Cervera, 2014	√	
Goldman, 2007	√	
Cohn, 2010	√	
Decarolis, 1994	√	
Faden, 1997	√	√
Farquharson, 1997	√	
Echavarri, 2014	√	
Julkunen, 1993	√	
Katano, 1996	√	√
Lockshin, 2012	√	
Madazli, 2013	√	
Matthiesen, 1999	√	√
Polzin, 1991	√	
Saccone, 2017	√	
Schei, 1995	√	
Shinozaki, 2016	√	
Yasuda, 1995	√	√
Milliez, 1991		√
Rix, 1992		√
CCA=20.83%		

Table S16. Overlapping between articles for the association between aPLs and CPR in ART.

	Gao, 2021	Tan, 2020
Birdsall, 1996	√	√
Denis, 1997		√
EI-Roeiy, 1987	√	
Chen, 2017	√	√
Chilcott, 2000	√	√
Hong, 2018	√	√
Sanmarco, 2007	√	√
Buckingham, 2006	√	
Lee, 2007	√	
Matsubayashi, 2006	√	
Ying, 2012	√	
Zhong, 2011	√	
Paulmyer, 2014	√	
Costa, 2012	√	
CCA=35.71%		

Table S17. Overlapping between articles for the association between aPLs and LBR in ART.

	Gao, 2021	Tan, 2020
Birdsall, 1996	√	√
Denis, 1997		√
EI-Roeiy, 1987	√	
Chen, 2017	√	√
Chilcott, 2000	√	√
Sanmarco, 2007	√	√
Buckingham, 2006	√	
Lee, 2007	√	
Paulmyer, 2014	√	
Hong, 2018	√	
CCA=40%		

Table S18. Overlapping between articles for the association between aPLs and Miscarriage rate in ART.

	Gao, 2021	Tan, 2020
Birdsall, 1996	√	√
Chen, 2017	√	√
Chilcott, 2000	√	√
Hong, 2018	√	√
Lee, 2007	√	
Paulmyer, 2014	√	
Sanmarco, 2007	√	√
Ying, 2012	√	
Zhong, 2011	√	
Denis, 1997		√
CCA=50%		

Table S19. Overlapping and non overlapping associations of included meta-analyses by CCA calculations.

Index	Author,Year	Quality	condition	Outcome	Number of include original studies	CCA	Decision
1	Bundhun, 2017	Low	SLE	Spontaneous abortion	4	0%	Yes
	He, 2020	Low			3		Yes
2	Bundhun, 2017	Low	SLE	PE	6	30.76%	No
	He, 2020	Low			5		No
	Dong (Yuan), 2019	Moderate			10		Yes
3	Dong (Dai), 2019	Low	SLE	GDM	5	30%	No
	He, 2020	Low			4		Yes
4	Bundhun, 2017	Low	SLE	Premature delivery	6	6.25%	Yes
	Wei, 2017	Moderate			6		Yes
	He, 2020	Low			6		Yes
5	Bundhun, 2017	Low	SLE	SGA	4	0%	Yes
	He, 2020	Low			4		Yes
6	Bundhun, 2017	Low	SLE	LBW	2	0%	Yes
	He, 2020	Low			3		Yes
7	Bundhun, 2017	Low	SLE	NICU	3	0%	Yes
	He, 2020	Low			2		Yes
8	Bundhun, 2017	Low	SLE	One minute Apgar < 7	3	0%	Yes
	He, 2020	Low			3		Yes
9	Upala, 2016	Moderate	pSS	Premature delivery	5	16.67%	No
	Geng, 2022	Moderate			9		Yes
10	Doprado, 2010	Moderate	aPLs	PE	12	26.92%	Yes
	Nassar, 2011	Low			21		No
11	Xu, 2022	Moderate	aPLs	IUGR	22	20.83%	Yes
	Nassar, 2011	Low			7		No
12	Tan, 2020	Low	aPLs	CPR in ART	6	20.83%	No
	Gao, 2021	Moderate			13		Yes
13	Tan, 2020	Low	aPLs	LBR in ART	5	40%	No
	Gao, 2021	Moderate			9		Yes

14	Tan, 2020	Low	aPLs	Miscarriage rate in ART	6	50%	No
	Gao, 2021	Moderate			9		Yes

Footnote: SLE, systemic lupus erythematosus; pSS, primary Sjögren syndrome; aPLs, antiphospholipid antibodies; pAPS, primary antiphospholipid syndrome; AMH, anti-mullerian hormone; PIH, pregnancy induced hypertension; PE, pre-eclampsia; SGA, small for gestational age; LBW, low birth weight; NICU, neonatal intensive care unit; GDM, gestational diabetes mellitus; IUGR, intrauterine growth retardation; PA, placental abruption; CPR, clinical pregnancy rate; LBR, live birth ratel; ART, assisted reproductive technology.

Table S20. The results of meta-analysis.

Conditions	Author, Year	Outcome	No. of studies	No. of participants	Summary estimates (95 CI)	I^2
SLE	Luo, 2020	AMH	11	937	SMD -0.79 (-1.41, -0.18)	94.6%
SLE	Bundhun, 2017	Spontaneous abortion	4	2974	RR 1.51 (1.26, 1.82)	38%
		PIH	4	498259	RR 1.99 (1.54, 2.56)	0%
		PE	4	288306	RR 1.91 (1.44, 2.53)	44%
		Premature birth	6	301593	RR 3.05 (2.56, 3.63)	47%
		SGA	4	475404	RR 1.69 (1.53, 1.88)	0%
		LBW	2	270467	RR 4.44 (3.53, 5.59)	0%
		Stillborn/neonatal death	3	2532	RR 1.70 (1.34, 2.16)	36%
		NICU	3	469344	RR 2.76 (2.27, 3.35)	0%
		One minute Apgar < 7	3	481919	RR 1.86 (1.51, 2.30)	0%
SLE	Wei, 2017	Premature birth	6	3690	RR 2.05 (1.72, 3.32)	66.5%
SLE	Dong (Dai), 2019	GDM	5	3432	RR 1.08 (0.49, 2.41)	76%
SLE	He, 2020	Spontaneous abortion	3	8792890	RR 4.70 (3.02, 7.29)	66%
		Total fetal loss	2	2414	RR 7.55 (4.75, 11.99)	0%
		PE	4	8794417	RR 3.38 (3.15, 3.62)	32%
		GDM	4	13993	RR 0.97 (0.57, 1.66)	80%
		Stillbirth	2	1499	RR 16.49 (2.95, 92.13)	0%
		IUGR	2	1448	RR 6.98 (0.33, 147.02)	79%
		Premature birth	6	8805962	RR 2.33 (1.78, 3.05)	94%
		SGA	4	20616	RR 2.50 (1.41, 4.45)	97%
		LBW	3	8501	RR 4.78 (3.65, 6.26)	59%
		NICU	2	4917	RR 2.79 (2.31, 3.37)	0%
		One minute Apgar < 7	3	2975	RR 2.47 (1.68, 3.62)	0%
SLE	Dong (Yuan), 2019	PE	10	9462549	RR 2.99 (2.31, 3.88)	76%
pSS	Upala, 2016	Total fetal loss	4	1537	OR 1.77 (1.28, 2.46)	0%
		Stillbirth	3	1951	OR 1.05 (0.37, 2.97)	0%
		Premature delivery	5	2217	OR 2.10 (0.59, 7.46)	59%
pSS	Geng, 2022	Spontaneous abortion	5	14515204	RR 8.85 (3.10, 25.26)	53%

		Premature birth	9	1284	RR 2.27 (1.46, 3.52)	63%
		LBW	5	959	RR 1.99 (1.34, 2.97)	42%
aPLs	Prado, 2010	PE	12	5704	OR 2.86 (1.37, 5.98)	68.7%
aPLs	Tan, 2022	CPR in ART	6	3214	RR 0.97 (0.91, 1.04)	32%
		LBR in ART	5	2943	RR 0.95 (0.81, 1.11)	61%
		Miascrriage rate in ART	6	1685	RR 1.22 (0.94, 1.58)	0%
aPLs	Gao, 2021	CPR in ART	13	3954	RR 0.95 (0.80, 1.13)	44%
		LBR in ART	9	3403	RR 1.01 (0.73, 1.39)	48%
		Miascrriage rate in ART	9	1702	RR 1.68 (1.24, 2.28)	7%
		Biochemical pregnancy rate in ART	2	469	RR 1.18 (0.57, 2.43)	0%
aPLs	Nassar, 2011	Total fetal loss	6	2798	OR 5.70 (2.67, 12.15)	43.3%
		PE	10	4676	OR 4.82 (1.99, 11.64)	77.9%
		PA	3	1044	OR 4.92 (0.86, 28.11)	3.8%
		IUGR	5	4676	OR 3.61 (1.79, 7.27)	0.2%
aPLs	Xu, 2022	IUGR	22	11745	OR 1.26 (1.12, 1.40)	16.1%
pAPS	Liu, 2019	Spontaneous abortion	3	396	RR 2.42 (1.46, 4.01)	39%
		Total fetal loss	5	1758	RR 1.33 (1.00, 1.76)	48%
		PIH	7	212887	RR 1.81 (1.33, 2.45)	40%
		PA	3	1532	RR 1.35 (0.78, 2.34)	0%
		Premature delivery	3	1520	RR 1.89 (1.52, 2.35)	0%
		SGA	3	1532	RR 1.38 (1.04, 1.82)	0%
		Stillborn/neonatal death	3	1551	RR 3.95 (1.98, 7.86)	0%
		NICU	3	211264	RR 3.35 (2.29, 4.89)	3%

Footnote: SLE, systemic lupus erythematosus; pSS, primary Sjögren syndrome; aPLs, antiphospholipid antibodies; pAPS, primary antiphospholipid syndrome; AMH, anti-mullerian hormone; PIH, pregnancy induced hypertension; PE, pre-eclampsia; SGA, small for gestational age; LBW, low birth weight; NICU, neonatal intensive care unit; GDM, gestational diabetes mellitus; IUGR, intrauterine growth retardation; PA, placental abruption; CPR, clinical pregnancy rate; LBR, live birth ratel; ART, assisted reproductive technology.
